# Palladium Nanoparticles Degrade Advanced Glycation End Products via Valosin‐Containing Protein Mediated Autophagy to Attenuate High‐Glucose/High‐Fat‐Induced Intervertebral Disc Degeneration

**DOI:** 10.1002/EXP.20230174

**Published:** 2025-01-17

**Authors:** Xiao Yang, Xiankun Cao, Xin Wang, Jiadong Guo, Yangzi Yang, Liqiang Lu, Pu Zhang, Huan Yang, Kewei Rong, Tangjun Zhou, Yongqiang Hao, Jie Zhao, Jingke Fu, Kai Zhang

**Affiliations:** ^1^ Shanghai Key Laboratory of Orthopedic Implants Department of Orthopedics Ninth People's Hospital Shanghai Jiao Tong University School of Medicine Shanghai China; ^2^ Department of Orthopedic Surgery Spine Center Changzheng Hospital Navy Medical University Shanghai China; ^3^ Institute of Electrochemical Energy Storage Helmholtz‐Zentrum Berlin für Materialien und Energie Berlin Germany; ^4^ The Second Clinical Medical College of Yunnan University of Traditional Chinese Medicine Kunming China

**Keywords:** advanced glycation end products, high‐glucose/high‐fat, intervertebral disc degeneration, nucleus pulposus, palladium nanoparticles

## Abstract

Intervertebral disc degeneration (IVDD) is a chronic musculoskeletal disorder causing lower back pain, imposing a considerable burden on global health. Hyperglycemia resulting from diabetes mellitus induces advanced glycation end products (AGEs) accumulation in nucleus pulposus cells, leading to IVDD. Mitigating AGEs accumulation is a novel promising strategy for IVDD management. In our study, palladium nanoparticles (Pd NPs) preferentially colocalized within the endoplasmic reticulum and efficiently degraded AGEs via valosin‐containing protein (VCP)‐mediated autophagy pathways. Pd NPs promoted the ATPase activity of VCPs, upregulated microtubule‐associated proteins 1A/1B light chain 3 (LC3) expression, and increased AGEs‐degrading autophagosome production. They ameliorated mitochondrial function, relieved endoplasmic reticulum stress, and counteracted the detrimental oxidative stress microenvironment in a high‐glucose/high‐fat‐induced nucleus pulposus cell degeneration model. Consequently, Pd NPs effectively rescued nucleus pulposus cell degeneration in vitro, restored disc height and partially recovered the degenerated phenotype of IVDD in vivo. We provide novel insights regarding IVDD management by targeting AGEs degradation, showing potential for clinical practice.

## Graphical abstract

Illustration of Pd NP‐mediated rescue of HGHF (or advanced glycation end products [AGEs])‐induced IVD degeneration by targeting AGEs degradation. Pd NPs promote the ATPase activity of VCP and upregulate autophagosome formation to degrade AGEs. Consequently, Pd NPs alleviate the oxidative and ER stress, protect the structure and function of mitochondria, and eventually facilitate IVDD mitigation. Created with BioRender.com (Agreement No. JU26337Q4Q).

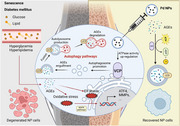

## 1 Introduction

Intervertebral disc degeneration (IVDD) is a prevalent chronic musculoskeletal disorder. It serves as the primary contributor to the onset of pain in the back or neck regions and radicular‐related discomfort [1]. Clinical treatment for symptomatic IVDD primarily relies on surgical intervention and conservative measures [2]. However, these therapies only relieve pain without reversing the pathophysiological function of the intervertebral disc (IVD). Thus, new treatments are urgently needed to attenuate IVDD and rely on a profound understanding of the molecular and pathogenetic mechanism of disc degeneration.

Increased production of reactive oxygen species (ROS) and various inflammatory cytokines are the major causes of the onset of disc degeneration [3]; however, a consensus regarding their origin has not been established. In recent studies, metabolic disorders caused by aging and diabetes mellitus (DM) were found to play a crucial role in IVDD progression. The disc microenvironment, with a low oxygen concentration and lack of vessel formation [4], is particularly susceptible to DM‐ and senescence‐associated vasculopathy and neuropathy. Furthermore, the disc microenvironment forms a hyperglycemic and hyperlipidemic microenvironment, leading to the generation of multiple detrimental oxidants within the IVD [5]. This increase in oxidants and inflammatory activation [6] accelerates IVDD development [7]. During this detrimental process, understanding of the mechanisms underlying an increase in cellular glucose uptake and accumulation of advanced glycation end products (AGEs) has garnered significant research interest [8]. AGEs are harmful oxidants produced via a nonenzymatic reaction between the carbonyl groups of reducing sugars and the free amine groups of proteins, nucleic acids, or lipids [9]. AGEs accumulation is a major biochemical hallmark of DM, resulting in the development of pathological phenotypes, including excessive oxidative stress, severe endoplasmic reticulum (ER) stress, and mitochondrial dysfunction of nucleus pulposus cells under hyperglycemic conditions [10]. Therefore, mitigating the accumulation of AGEs may be a promising strategy to relieve IVDD.

Palladium nanoparticles (Pd NPs) have been used as catalysts to participate in chemical reactions. For instance, Chen et al. used Pd NPs as efficient depropargylation catalysts to transform metabolically incorporated *N*‐(propargyloxycarbonyl) neuramic acid into neuramic acid [11]. Furthermore, Pd(0) NPs catalyzed the in situ synthesis of a BODIPY dye via the Suzuki–Miyaura reaction in vitro, leading to fluorescent labelling of mammalian cells [12]. In this study, we aimed to investigate the potential therapeutic role of Pd NPs in mitigating IVDD by targeting the accumulation of AGEs via valosin‐containing protein (VCP)‐mediated autophagy pathways. We aimed to demonstrate the efficacy of Pd NPs in rescuing IVDD induced by high‐glucose/high‐fat (HGHF) and AGEs using both in vitro nucleus pulposus cells and in vivo animal models. Additionally, we sought to elucidate the mechanisms underlying Pd NP‐mediated mitigation of IVDD. Overall, we aimed to highlight the potential of Pd NPs as efficient and biocompatible therapeutic agents for IVDD treatment. This is the first study to elucidate the use of Pd NPs in upregulating the VCP‐mediated autophagy of AGEs, highlighting the potential of Pd NPs as highly efficient and biocompatible therapeutic agents for attenuating IVDD.

## 2 Results

### 2.1 AGEs Accumulation in Degenerated IVDs of Patients and *db/db* Mice

**FIGURE 1:**
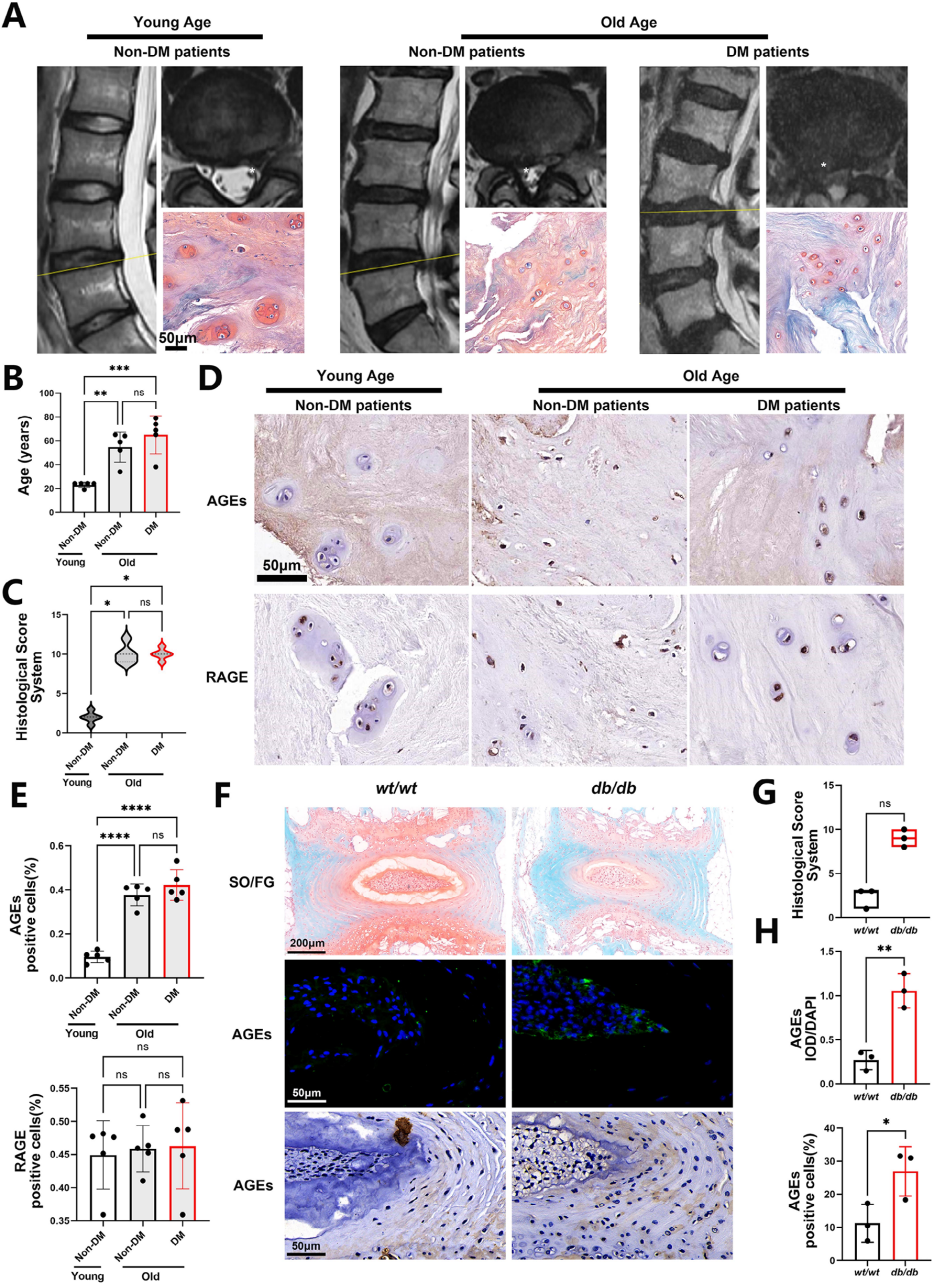
AGEs accumulate in the degenerated IVDs of patients and *db/db* mice. (A) Representative magnetic resonance images (MRI) and Safranin O‐fast green (SO/FG) stain images of clinical intervertebral disc (IVD) samples. *Nucleus pulposus region. (B) Quantification of the ages of patients. (C) Histological score of IVD samples. (D) Immunohistochemistry analysis of IVD samples. (E) Quantification of advanced glycation end products (AGEs) and AGE receptor (RAGE)‐positive cells shown in (D). (F) SO/FG stain, immunofluorescence, and immunohistochemistry analysis of the lumbar spines of *wt/wt* and *db/db* mice. (G) Histological score of sections shown in (F). (H) Integrated optical density (IOD)/4′,6‐diamidino‐2‐phenylindole (DAPI) quantification of AGEs and positive cells shown in (F). Data are presented as the mean ± standard deviation (SD) from three or five replicates. ^*^ *p *< 0.05, ^**^ *p *< 0.01, ^***^ *p *< 0.001, and ^****^ *p *< 0.0001.

We divided clinical IVD samples obtained from 15 patients into three groups (*n* = 5). The control group included young patients (19 to 24 years) without DM. The older age group included older patients (34 to 65 years) without DM. The DM group included older patients (38 to 79 years) with DM (Figure 1A,B). Severe IVDD was observed in older patients with or without DM (Figure 1C and Figure S1). AGEs accumulation was significantly increased in the older and DM groups (Figure 1D,E), regardless of the presence of DM. This result suggested that the IVDD was accompanied by the accumulation of AGEs. However, the expression of the primary AGE receptor (RAGE) showed no differences in all groups (Figure 1D,E). These findings indicate that the intervertebral disc (IVD) is vulnerable to elevated levels of AGEs but not to RAGE itself. We also collected disc samples from *db/db* mice (global knockout of *Lepr* to induce DM in mice, *n* = 3) to further confirm the involvement of AGEs in IVDD. Compared to those in the *wt/wt* mice, marked severe degeneration and distinct AGEs accumulation were seen in the discs of the *db/db* mice (Figure 1F–H). These results substantiated the involvement of AGEs accumulation in disc degeneration, especially in DM‐related IVDD in *db/db* mice. Thus, eliminating AGEs may be an effective management strategy for the treatment of IVDD.

### 2.2 Characterization of Pd NPs and Their Endocytosis in Nucleus Pulposus Cells for Degradation of AGEs

**FIGURE 2:**
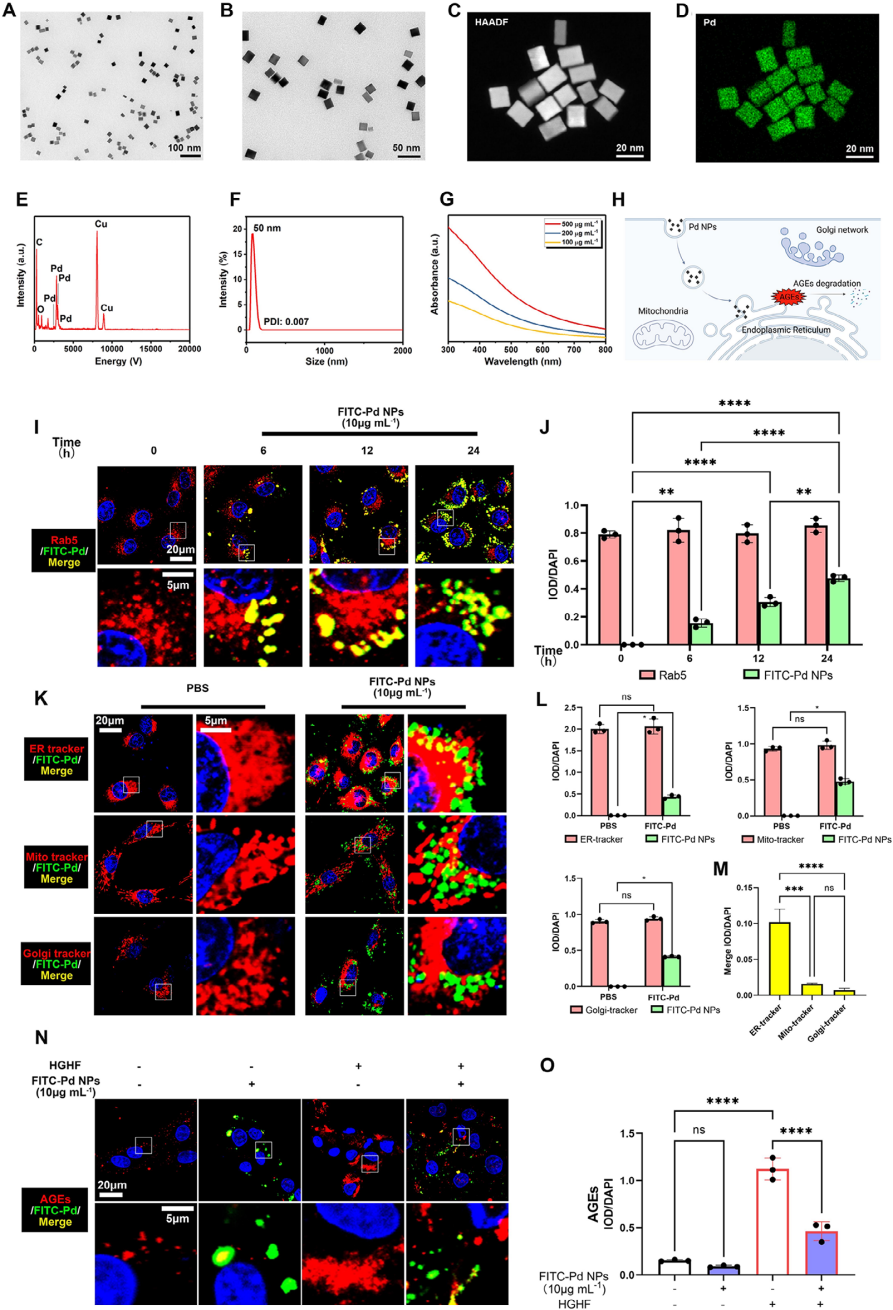
Characterization of Pd NPs and their endocytosis in nucleus pulposus cells to degrade AGEs. (A,B) Representative transmission electron microscopy (TEM) images of palladium nanoparticles (Pd NPs). (C–E) High‐angle annular dark field, elemental mapping images, and energy‐dispersive X‐ray spectroscopy of Pd NPs. (F) The hydrodynamic particle size distribution of Pd NPs. (G) Ultraviolet–visible absorption spectroscopy of Pd NPs. (H) Illustration of the endocytosis of Pd NPs in nucleus pulposus cells created with BioRender.com (Agreement No. FV26JP5S0G). (I) Confocal laser scanning microscopy (CLSM) of nucleus pulposus cells after treatment with fluorescein isothiocyanate (FITC)‐Pd NPs (10 µg mL^−1^; 0, 6, 12, and 24 h). (J) The IOD/DAPI quantification of Rab5 and FITC‐Pd NPs is shown in I. (K) CLSM test of endoplasmic reticulum (ER)‐, mitochondrial (Mito)‐, and Golgi apparatus (Golgi)‐trackers in nucleus pulposus cells stimulated with FITC‐Pd NPs (0 or 10 µg mL^−1^) for 24 h. (L) IOD/DAPI quantification of the ER‐, Mito‐, and Golgi‐trackers shown in (K). (M) Merged IOD/DAPI quantification shown in (K). (N) CLSM of AGEs in nucleus pulposus cells with FITC‐Pd NPs, high‐glucose/high‐fat (HGHF), or both conducted for 24 h. (O) Quantification of IOD/DAPI of AGEs shown in (N). Data are presented as the mean ± standard deviation (SD) derived from three replicates. ^*^ *p *< 0.05, ^**^ *p *< 0.01, ^***^ *p *< 0.001, and ^****^ *p *< 0.0001.

Pd NPs were prepared as previously described with some modifications [13]. Transmission electron microscopy (TEM) images (Figure 2A,B) showed the as‐prepared Pd NPs were monodispersed and uniform. The homogeneity of Pd NPs was further confirmed with high‐angle annular dark field scanning TEM and energy‐dispersive X‐ray spectroscopy (Figure 2C–E). Dynamic light scattering indicated that the hydrodynamic diameter of Pd NPs was 50 nm with restricted size distribution (Figure 2F). The Pd NPs can be dispersed homogeneously in water and maintain stability after dispersion in phosphate‐buffered saline (PBS) for 14 days (Figure S2), facilitating their biomedical application in vivo. The Pd NPs showed absorption over a wide range (300–800 nm) based on the ultraviolet‐visible absorption spectrum, with absorption increasing in a Pd NP dose‐dependent manner (Figure 2G).

The as‐prepared Pd NPs were labeled with fluorescein isothiocyanate (FITC) to obtain FITC‐Pd NPs. The uptake of FITC‐Pd NP was monitored in nucleus pulposus cells using confocal laser scanning microscopy (CLSM; Figure 2H). FITC‐Pd NPs were internalized into nucleus pulposus cells and colocalized with the endocytosis marker Rab5 (Figure 2I). In addition, the green fluorescence intensity of FITC‐Pd NPs in cells increased over time (0, 6, 12, and 24 h; Figure 2I,J). Notably, more FITC‐Pd NPs were colocalized with the ER‐tracker (GFP‐labeled glibenclamide to mark K^+^ ATP channels in the ER membrane) than with the mitochondrial (Mito)‐tracker (GFP‐labeled mildly thiol‐reactive chloromethyl to mark mitochondria) and the Golgi apparatus (Golgi)‐tracker (GFP‐labeled ceramide to mark the Golgi apparatus; Figure 2K–M). These results suggest the possible interaction of Pd NPs with the ER. Further CLSM observations showed that FITC‐Pd NPs were colocalized with AGEs (Figure 2N), and Pd NPs could significantly repress AGEs accumulation in nucleus pulposus cells under HGHF conditions (*p *< 0.0001, Figure 2N,O). Further investigation of the interaction between the Pd NPs and AGEs was performed with commercially purchased AGEs (bovine serum albumin (BSA)‐AGEs labeled with Cy3 stain; Cy3‐AGEs). The intracellular content of extrinsic Cy3‐AGEs increased over time (0, 8, 12, and 24 h; Figure S3). Importantly, FITC‐Pd NPs colocalized with these proteins and reduced the intensity of extrinsic Cy3‐AGEs (Figure S3). By contrast, no colocalization of FITC‐Pd NPs with RAGE was observed in HGHF‐stimulated nucleus pulposus cells (Figure S4A,B), demonstrating that Pd NPs exerted effects primarily via AGEs but not in conjunction with RAGE.

### 2.3 Pd NPs Degrade AGEs via the VCP‐mediated Autophagy Pathway

**FIGURE 3:**
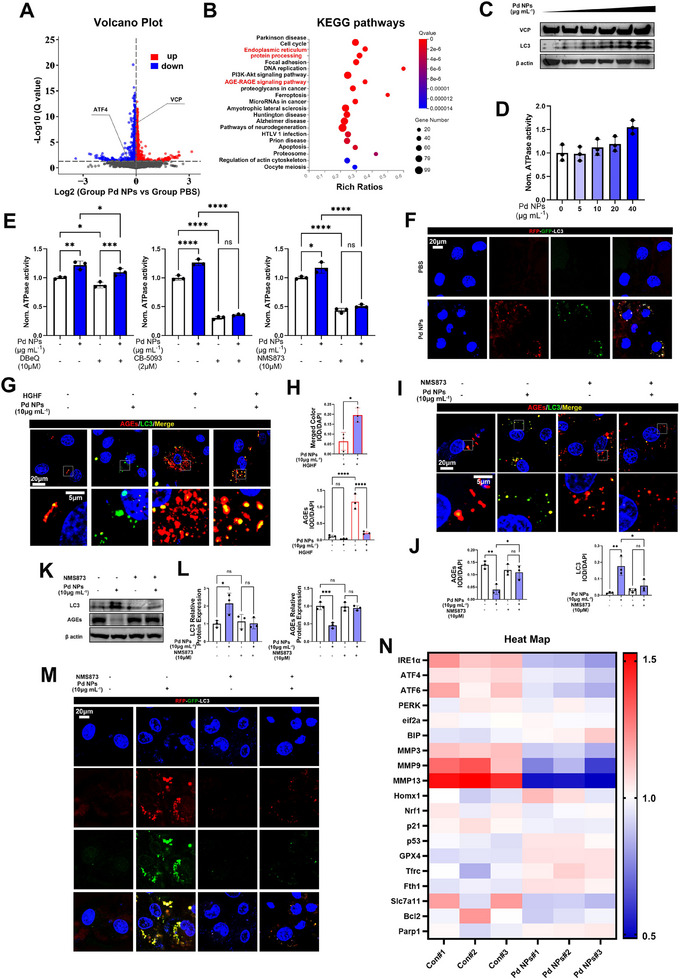
Pd NPs degrade AGEs via the VCP‐mediated autophagy pathway. (A) Volcano plot analysis of nucleus pulposus cells stimulated with Pd NPs (0 or 10 µg mL^−1^; 24 h). (B) Kyoto Encyclopedia of Genes and Genomes pathway analysis of nucleus pulposus cells stimulated with Pd NPs (0 or 10 µg mL^−1^; 24 h). (C) Western blotting (WB) analysis of microtubule‐associated proteins 1A/1B light chain 3 (LC3) and valosin‐containing protein (VCP) in nucleus pulposus cells treated with Pd NPs (0, 0.625, 1.25, 2.5, 5, or 10 µg mL^−1^; 24 h). (D) ATPase activity assay of VCP upon adding Pd NPs (0, 2.5, 5, 10, or 20 µg mL^−1^). (E) ATPase activity assay of VCP upon adding Pd NPs (10 µg mL^−1^) with VCP inhibitors: 10 µM DBeQ, 2 µM CB‐5083, or 10 µM NMS873. (F) CLSM of GFP‐RFP‐LC3 in nucleus pulposus cells after treatment with Pd NPs (0 or 10 µg mL^−1^; 24 h). (G) CLSM of AGEs and LC3 in nucleus pulposus cells after treatment with HGHF, Pd NPs, or both (10 µg mL^−1^; 24 h). (H) IOD/DAPI quantification of AGEs and merged color images shown in (G). (I) CLSM of AGEs and LC3 in nucleus pulposus cells upon adding Pd NPs (10 µg mL^−1^) with and without 10 µM NMS873 for 24 h. (J) IOD/DAPI quantification of AGEs and LC3 shown in (I). (K) WB analysis of LC3 and AGEs in nucleus pulposus cells upon adding Pd NPs (10 µg mL^−1^; 24 h) with and without 10 µM NMS873. (L) Semi‐quantification of the greyscale value of LC3 and AGEs shown in (K). (M) CLSM of GFP‐RFP‐LC3 in nucleus pulposus cells upon adding Pd NPs (10 µg mL^−1^; 24 h) with and without 10 µM NMS873. (N) Heat map analysis of nucleus pulposus cells with Pd NPs (0 or 10 µg mL^−1^; 24 h). Data are presented as the mean ± standard deviation (SD) derived from three replicates. ^*^ *p *< 0.05, ^**^ *p *< 0.01, ^***^ *p *< 0.001, and ^****^ *p *< 0.0001.

We then explored the pathophysiology of Pd NPs using RNA sequence analysis. The significantly differentially expressed genes are shown in the volcano plot (Figure 3A). The ER protein processing and the AGEs‐RAGE signaling pathway were involved in this process based on the KEGG pathway analysis (Figure 3B). Given that Pd NPs preferentially colocalized with ER and AGEs, the involvement of ER protein processing and the AGEs‐RAGE signaling pathway prompted us to hypothesize that the interaction of Pd NPs with AGEs may be related to the located proteins in the primary cellular protein processing factory, namely the ER. According to the RNA sequence analysis, VCP was involved in the ER protein processing pathway. We, thus, hypothesized that Pd NP‐mediated AGEs degradation may depend on the VCP ATPase. Although the increased concentration of Pd NPs did not change the protein expression of VCP, upregulation of the expression of LC3 (autophagy and autophagy‐related processes marker) and the ATPase activity of VCP was observed (Figure 3C,D). These results demonstrated that Pd NPs participated in VCP‐mediated autophagy.

VCP inhibitors such as DBeQ, CB‐5093, and NMS873 were employed to further confirm the mechanism of action, and these compounds could distinctly abate ATPase activity upregulation by Pd NPs (Figure 3E). Then, the RFP‐GFP‐LC3 kit was used to further analyze the Pd NP‐promoted, LC3‐labeled autophagosome production (LC3 proteins were labeled with GFP and RFP both in the autophagosomes; GFP is acid‐sensitive once the autophagosomes are fused to acidic autolysosome, and the corresponding fluorescence is quenched; Figure 3F and Figure S5A). Notably, LC3‐labeled autophagosome and AGEs colocalization increased after Pd NP treatment, further decreasing AGEs accumulation under conditions involving HGHF, as shown using CLSM (Figure 3G,H). These observations suggested that Pd NPs upregulated LC3‐mediated autophagy toward AGEs and that this process relied on the ATPase activity of VCP. Furthermore, the Pd NP‐mediated LC3 increase and AGEs decrease in nucleus pulposus cells were significantly abated when NMS873 was used to inhibit VCP (Figure 3I,J). Moreover, western blotting (WB) analysis corroborated these findings demonstrating that NMS873 countered the Pd NP‐mediated increase in LC3 and a decrease in AGEs (Figure 3K,L). NMS873 inhibited the ATPase activity of VCP and LC3‐labeled autophagosome production (Figure 3M and S5B), further inhibiting autophagy. Therefore, Pd NPs promoted the ATPase activity of VCP, thereby facilitating LC3‐mediated autophagy targeted toward AGEs.

In addition, the gene heat map showed that the expression of genes encoding matrix metalloproteinase 9 and 13 *(MMP9* and *13*) decreased, whereas that of the genes encoding glutathione peroxidase 4 *(GPX4)* and heme oxygenase 1 *(Homx1*, abbreviated as *HO‐1)* increased after Pd NP treatment (Figure 3N). Therefore, Pd NPs may also be involved in the oxidative and inflammatory stress in nucleus pulposus cells.

### 2.4 Pd NPs Counteract HGHF (or AGEs)‐Induced Oxidative and ER Stress In Vitro

**FIGURE 4:**
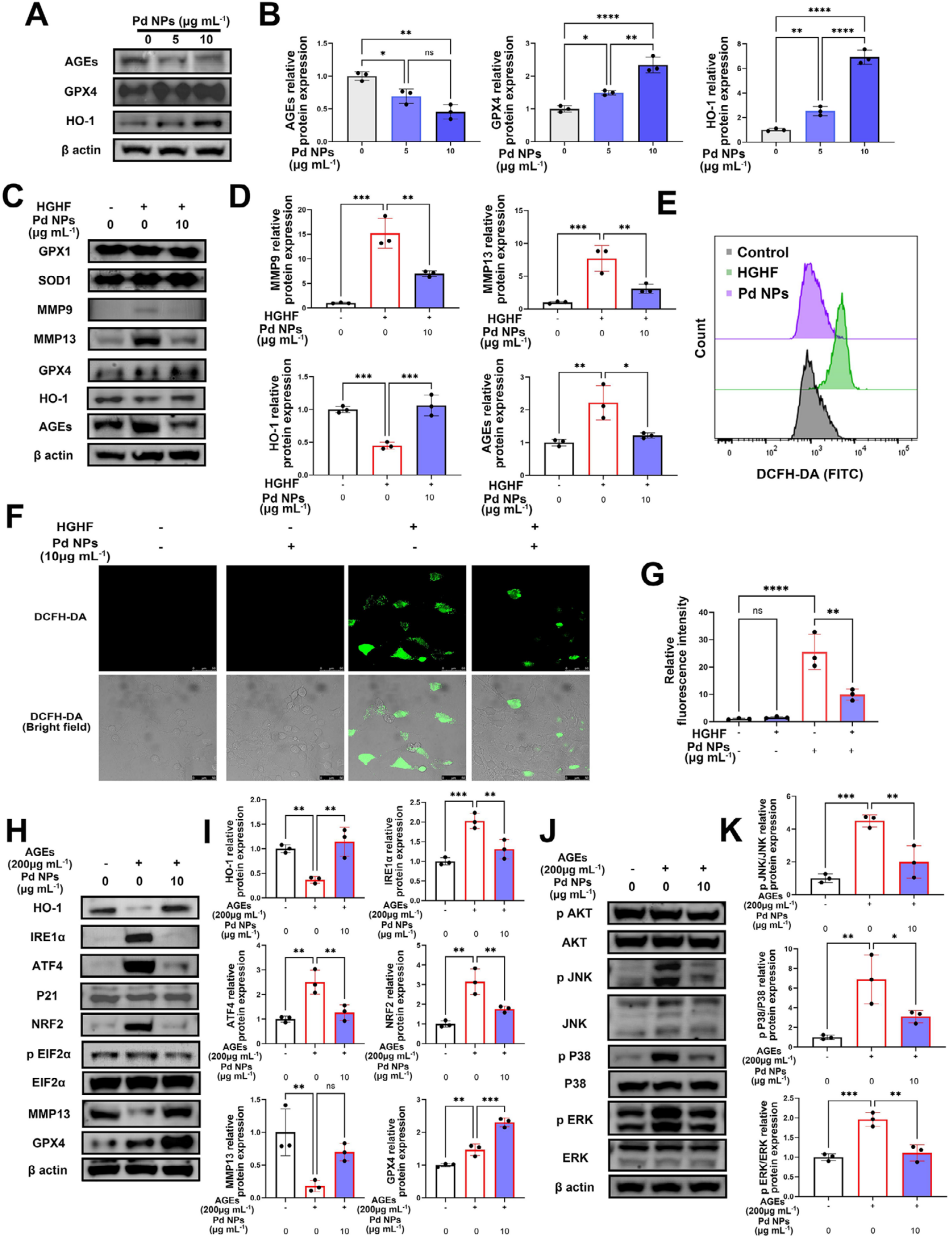
Pd NPs counteract HGHF (or AGEs)‐induced oxidative and ER stress in vitro (A) WB analysis of AGEs, glutathione peroxidase 4 (GPX4), and heme oxygenase 1 (HO‐1) in nucleus pulposus cells stimulated with Pd NPs (0, 5, or 10 µg mL^−1^; 24 h). (B) Semi‐quantification of the greyscale values in AGEs, GPX4, and HO‐1 shown in A. (C) WB analysis of GPX1, superoxide dismutase type 1 (SOD1), matrix metalloproteinase (MMP) 9, MMP13, GPX4, HO‐1, and AGEs in nucleus pulposus cells stimulated with HGHF and Pd NPs (0 or 10 µg mL^−1^; 24 h). (D) Semi‐quantification of the values of MMP9, MMP13, HO‐1, and AGEs shown in (C). (E) Flow cytometry analysis of nucleus pulposus cells stained with 2′,7′‐dichlorofluorescein diacetate (DCFH‐DA) and stimulated with HGHF and Pd NPs (0, 10 µg mL^−1^; 24 h). (F) Immunofluorescence analysis of DCFH‐DA in nucleus pulposus cells stimulated with HGHF and Pd NPs (0 or 10 µg mL^−1^; 24 h). (G) Quantification of the relative fluorescence intensity of DCFH‐DA shown in (F). (H) WB analysis of HO‐1, inositol requiring protein‐1α (IRE1α), activating transcription factor‐4 (ATF4), P21, nuclear factor erythroid 2‐related factor 2 (NRF2), phospho(p)‐EIF2α, EIF2α, MMP13, and GPX4 in nucleus pulposus cells treated with AGEs (200 µg mL^−1^) and Pd NPs (0 or 10 µg mL^−1^; 24 h). (I) Semi‐quantification of the greyscale values in HO‐1, IRE1α, ATF4, NRF2, MMP13, and GPX4 shown in (H). (J) WB analysis of phospho‐protein kinase B (p‐AKT), AKT, phospho‐c‐Jun‐amino‐terminal kinase (p‐JNK), JNK, p‐P38, P38, phospho‐extracellular signal‐regulated kinases (p‐ERK), and ERK in nucleus pulposus cells stimulated with AGEs and Pd NPs (0 or 10 µg mL^−1^; 24 h). (K) Semi‐quantification of the greyscale values in p‐JNK/JNK, p‐P38/P38, and p‐ERK/ERK shown in (J). Data are presented as the mean ± standard deviation (SD) derived from three replicates. ^*^ *p *< 0.05, ^**^ *p *< 0.01, ^***^ *p *< 0.001, and ^****^ *p *< 0.0001.

WB analysis showed that Pd NPs significantly decreased the AGEs levels (Figure 4A,B). GPX4 and HO‐1 levels showed a concentration‐dependent increase after administration of Pd NPs (Figure 4A,B). Nucleus pulposus cells were treated with HGHF to further investigate the role of Pd NPs toward oxidative stress and inflammation, and the AGEs, MMP9, and MMP13 levels in cells were evaluated via WB analysis. HGHF treatment significantly enhanced AGEs, MMP9, and MMP13 (*p *< 0.0001) levels, and Pd NPs downregulated these levels efficiently in nucleus pulposus cells (Figure 4C,D). Moreover, under HGHF conditions, the administration of Pd NPs led to the restoration of the HO‐1 levels, demonstrating the re‐establishment of antioxidant abilities in nucleus pulposus cells. Pd NP treatment significantly depleted excessive ROS and counteracted the oxidative stress in nucleus pulposus cells (Figure 4E–G and Figure S6). These results confirmed that Pd NPs alleviated oxidative stress and inflammation in HGHF‐stimulated nucleus pulposus cells.

We assumed that Pd NPs mediated AGEs degradation and alleviated oxidative stress and inflammation, which may also affect ER stress in HGHF‐stimulated nucleus pulposus cells. The gene heat map (Figure 3N) revealed that the expression of genes involved in ER protein processing, such as *inositol requiring protein‐1α (IRE1α)* and *activating transcription factor‐4 (ATF4)*, decreased after Pd NP treatment. WB analysis also showed that Pd NPs reduced IRE1α and ATF4 (ER stress markers) levels and restored the HO‐1 levels in the AGEs‐stimulated nucleus pulposus cells (Figure 4H,I). Moreover, Pd NPs inactivated the mitogen‐activated protein kinase (MAPK) signaling pathway by downregulating the phosphorylated c‐Jun‐amino‐terminal kinase (JNK) and P38 pathways and inactivated the extracellular signal‐regulated kinases 1 and 2 (ERK1/2) and protein kinase B (AKT) pathways in AGEs‐treated nucleus pulposus cells (Figure 4J,K). Inactivation of this signaling pathway typically reduces oxidative stress, inflammation, and AGEs accumulation in a positive feed‐forward loop, elucidating the mechanism underlying Pd NP‐mediated IVDD mitigation.

### 2.5 Pd NPs Protect the Structure and Function of Mitochondria in HGHF (or AGEs)‐Induced Nucleus Pulposus Cell Degeneration In Vitro

**FIGURE 5:**
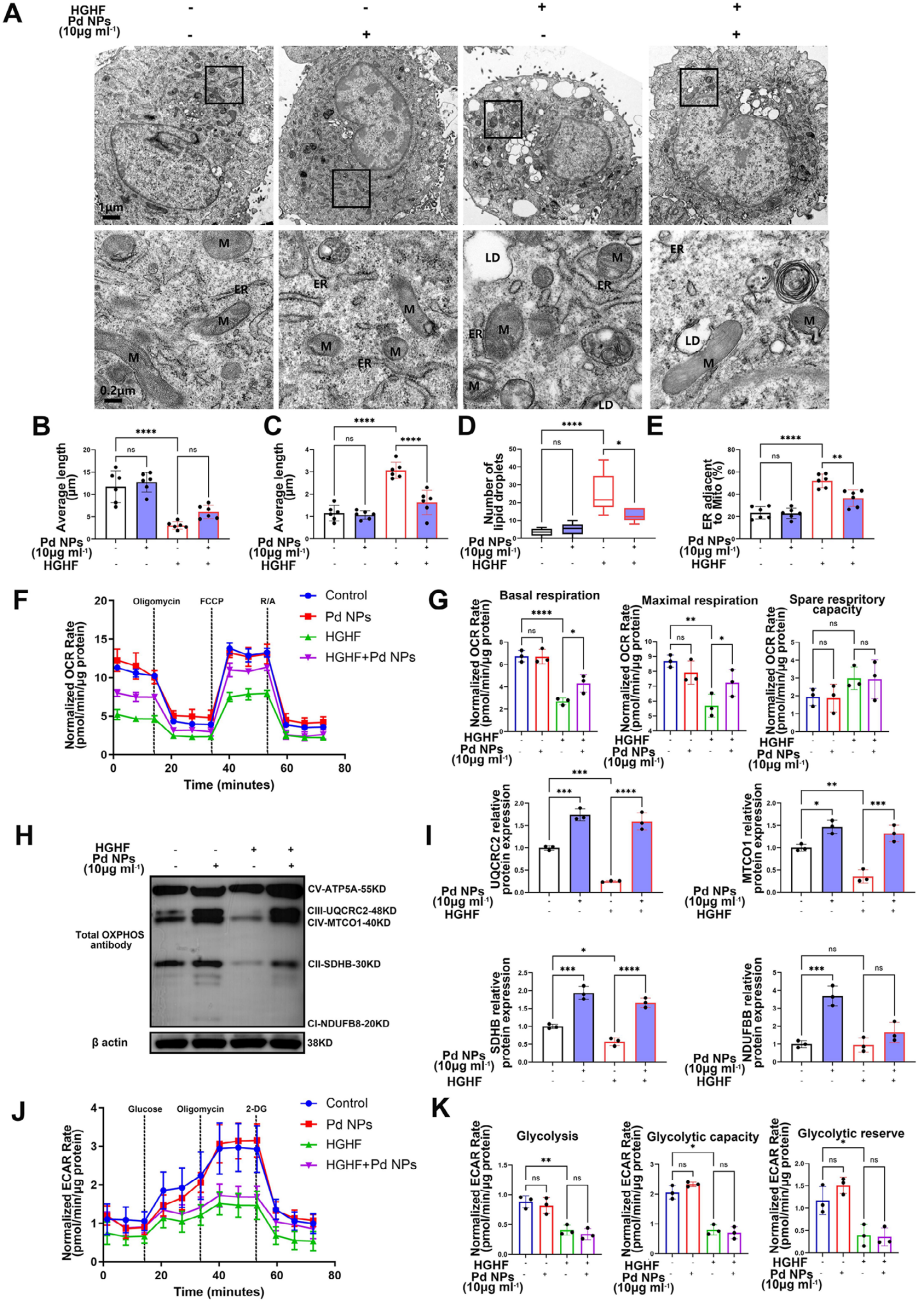
Pd NPs protect the structure and function of mitochondria in HGHF (or AGEs)‐mediated nucleus pulposus cell degeneration in vitro. (A) TEM analysis of nucleus pulposus cells treated with HGHF and Pd NPs (0 or 10 µg mL^−1^; 24 h). (B,C) Quantification of the mitochondrial length and width shown in (A). (D) Lipid droplet quantification shown in (A). (E) The percentage of ER adjacent to mitochondria shown in (A). (F) Oxygen consumption rate (OCR) assay of nucleus pulposus cells treated with HGHF and Pd NPs (0 or 10 µg mL^−1^; 24 h). (G) Quantification of basal and maximal respiration and spare respiratory capacity shown in (F). (H) WB analysis of oxidative phosphorylation (OXPHOS) proteins in nucleus pulposus cells treated with HGHF and Pd NPs (0 or 10 µg mL^−1^; 24 h) (I) Semi‐quantification of the grayscale values in cytochrome b‐c1 complex subunit 2, cytochrome c oxidase subunit 1, succinate dehydrogenase [ubiquinone] iron‐sulfur subunit, and NADH dehydrogenase [ubiquinone] 1 beta subcomplex subunit 8 shown in (H). (J) Extracellular acidification rate assay of nucleus pulposus cells treated with HGHF and Pd NPs (0 or 10 µg mL^−1^; 24 h). (K) Quantification of glycolysis, glycolytic capacity, and glycolytic reserve shown in J. Data are presented as the mean ± standard deviation (SD) derived from three or six replicates. ^*^ *p *< 0.05, ^**^ *p *< 0.01, ^***^ *p *< 0.001, and ^****^ *p *< 0.0001.

The structure and function of mitochondria were, thus, evaluated in the HGHF model in vitro. Mitochondrial structures changed with decreased length and increased width in HGHF‐induced nucleus pulposus cells (Figure 5A–C). Moreover, the lipid droplets and the percentage of mitochondria‐associated ER membrane [14] significantly increased in HGHF‐stimulated nucleus pulposus cells (Figure 5A–E). Notably, the administration of Pd NPs distinctly ameliorated the changes in mitochondrial structure induced by HGHF (Figure 5A–E). The seahorse assay was then used to analyze the mitochondrial function. The reduced oxygen consumption rate (OCR, mitochondrial respiration), particularly basal and maximal respiration, in HGHF‐stimulated nucleus pulposus cells was substantially recovered by Pd NPs (Figure 5F,G). Moreover, Pd NPs upregulated oxidative phosphorylation (OXPHOS) protein levels, including cytochrome b‐c1 complex subunit 2, cytochrome c oxidase subunit 1, succinate dehydrogenase [ubiquinone] iron‐sulfur subunit, and NADH dehydrogenase [ubiquinone] 1 beta subcomplex subunit 8 in HGHF‐stimulated nucleus pulposus cells (Figure 5H,I). Additionally, the mitochondrial function of nucleus pulposus cells was evaluated after the stimulation with BSA‐AGEs. A similar protective effect on mitochondrial function was observed after the Pd NP treatment (Figures S7 and S8). The glycolytic function of nucleus pulposus cells was then evaluated using the extracellular acidification rate assay. Pd NPs recovered the glycolytic capacity, glycolysis, and glycolytic reserves of AGEs‐stimulated nucleus pulposus cells. By contrast, they did not influence the glycolytic function of HGHF‐stimulated nucleus pulposus cells (Figure 5J,K).

### 2.6 Pd NPs Mitigate AGEs‐Induced IVDD via VCP‐Mediated Autophagy in Rat Tails

**FIGURE 6:**
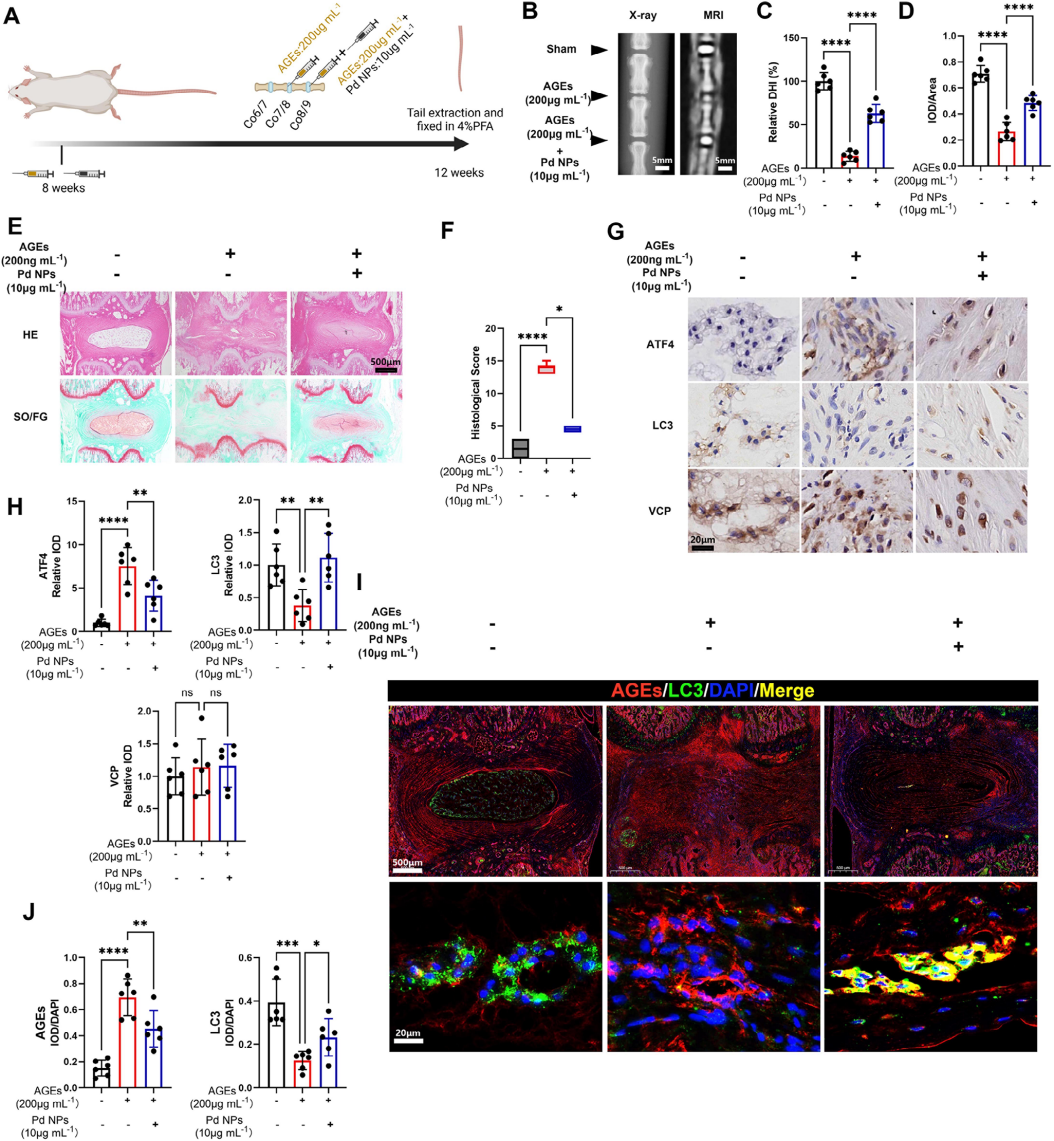
Pd NPs mitigate AGEs‐induced IVDD via VCP‐mediated autophagy in rat tails. (A) Schematic diagram demonstrating the development of the IVDD model in rats induced by AGEs and treatment with Pd NPs. All rats (*n* = 6) were injected with AGEs (200 µg mL^−1^) at coccyx vertebrae (Co)7/8 and Co8/9. Co8/9 was rescued with Pd NPs (10 µg mL^−1^). Images were created with BioRender.com (Agreement No. SP26JP5I26). (B) X‐ray and magnetic resonance imaging (MRI) analysis of the IVDD model focused on the area of operation. (C) Quantification of the disc height index (DHI) shown in (B). (D) IOD/DAPI quantification of the high‐intensity T2 signal shown in (B). (E) H&E and SO/FG staining of the tails after surgery. (F) Histological score quantification of the sections shown in (F). (G) Immunohistochemistry analysis of ATF4, LC3, and VCP. (H) IOD quantification of the relative ATF4, LC3, and VCP levels described in (G). (I) Immunofluorescence analysis of AGEs and LC3. (J) IOD/DAPI quantification of AGEs and LC3 shown in (I). Data are presented as the mean ± standard deviation (SD) derived from six replicates. ^*^ *p *< 0.05, ^**^ *p *< 0.01, ^***^ *p *< 0.001, and ^****^ *p *< 0.0001.

The efficacy and the mechanism of the Pd NPs were explored in a rat IVDD model. This IVDD model was established by the administration of AGEs via rat tails (Figure 6A). Severe disc degeneration in rat tails was confirmed by the significantly decreased disc height index (DHI) (Figure 6B,C). The DHI was distinctly recovered by administering Pd NPs, suggesting mitigation of disc degeneration in vivo (Figure 6B,C). The IOD of the magnetic resonance imaging (MRI) T2 signal was significantly increased, further verifying that Pd NPs mediated recovery of IVDD in rat tails (Figure 6B,D). Furthermore, hematoxylin and eosin (H&E) and Safranin O‐fast green (SO/FG) staining of disc tissues showed IVDD alleviation after Pd NP treatment, further substantiated by the histological scoring systems (Figure 6E,F). Local usage of Pd NPs showed excellent in vivo biocompatibility. The biochemistry analyses showed that the levels of albumin (ALB), total bilirubin (TBil), alanine aminotransferase (ALT), aspartate aminotransferase (AST), glucose (GLU), gamma‐glutamyltransferase (γ‐GT), blood urea nitrogen (BUN), and creatinine (Scr) in rat serum were nearly identical in the PBS and Pd NPs groups (Figures S9A). Moreover, the histological analyses of the major organs, including the heart, liver, spleen, lung, and kidney, showed no distinct abnormalities after treatment with Pd NPs (Figures S9B). These results indicated that local intra‐intervertebral injection of Pd NPs at tested dosages was safe for in vivo IVDD treatment. Immunohistochemistry showed that Pd NPs reduced the IOD intensity of ATF4 (an ER stress marker) and recovered LC3 expression (an autophagy marker) in the AGEs‐induced IVDD model (Figure 6G,H). However, VCP expression remained the same. The immunofluorescence assay indicated the accumulation of AGEs and depletion of LC3 in the IVDD group (Figure 6I,J). However, Pd NPs effectively restored LC3 expression and downregulated the excessive AGEs (Figure 6I,J). Moreover, LC3 was colocalized with AGEs within a focused area of IVD (Figure 6I), demonstrating that LC3‐mediated autophagy played a role in Pd NP‐mediated degradation of excessive AGEs. This Pd NP‐mediated depletion of AGEs contributed to the mitigation of IVDD under in vivo conditions.

## 3 Discussion

The unique microenvironment of IVD features a lack of oxygen and vessels [15], rendering it more susceptible to systematic metabolic diseases, such as DM. DM is characterized by insulin resistance, hyperglycemia, and AGEs accumulation [16]. Hyperglycemia [17] and neovascularization [18] resulting from DM impair disc metabolism through AGEs accumulation. In this study, clinical samples from patients with DM showed severe IVDD. In addition, AGEs accumulation was distinctly observed in the disc tissues of these patients, further confirmed by in vivo results from *db/db* murine samples. Excessive production of AGEs was observed in vitro in nucleus pulposus cells stimulated with HGHF. Hence, targeting the clearance of AGEs is a promising strategy for treating DM‐associated IVDD [19]. Existing therapeutic nanosystems primarily rely on mitigating the AGEs‐induced increase in ROS [20], the clearance of AGEs by inhibiting AGEs synthesis [21], or interference with the interaction between AGEs and RAGE [22]. An efficient nanosystem targeting the degradation of AGEs is yet to be reported. In this study, we report for the first time that biocompatible Pd NPs could alleviate IVDD by degrading AGEs. Moreover, we explored the mechanism of Pd NPs. The findings demonstrate that Pd NPs promoted the ATPase activity of VCP and activated the LC3‐mediated autophagy pathways to degrade AGEs. Moreover, they could relieve ER stress and recover mitochondrial function, working synergistically to mitigate HGHF‐ and AGEs‐induced IVD degeneration in nucleus pulposus cells and animal models. This study offers a novel strategy to mitigate IVDD by targeting AGEs degradation via Pd NPs and provides insights for developing novel therapeutic measures for IVDD.

Pd NPs were endocytosed into nucleus pulposus cells and preferentially colocalized with the ER. ER is a protein processing factory that maintains the inner homeostasis of cells [23]. Notably, Pd NPs tended to colocalize with intracellular AGEs and significantly decreased the levels of AGEs in HGHF‐stimulated nucleus pulposus cells. Hence, Pd NPs degraded AGEs by modulating the ER functions. VCP is a ubiquitously expressed ATPase located at the ER membrane and acts in conjunction with the ubiquitin‐proteasome system (UPS) [24]. VCP is critical in controlling UPS‐mediated protein processing in ER‐related protein degradation, autophagy, and apoptosis [25]. RNA sequence analysis showed that VCP was involved in the ER protein processing pathway. Furthermore, it is an ATP‐dependent protein located in the ER membrane, which plays a critical role in ubiquitin‐dependent protein quality control [26] and autophagy [27]. In this study, although Pd NPs did not change the VCP protein expression, they upregulated the expression of LC3 (an important autophagy marker) and the ATPase activity of VCP. This process could be significantly abated by the VCP inhibitor NMS873, confirming that Pd NPs participated in the VCP‐mediated autophagy targeted toward AGEs in nucleus pulposus cells. The LC3‐labeled autophagosomes were observed to accumulate and engulf AGEs, subsequently merging with lysosomes to form autolysosomes and digest AGEs.

AGEs, originating from dietary sources and cellular metabolism, are considered detrimental to health [28]. AGEs constantly interact with RAGE to exert proinflammatory effects and aggravate oxidative stress [29]. The interaction of AGEs with RAGE initiates various signaling pathways, such as [30] the inflammatory MAPK signaling, AKT, ERK1/2, NADPH oxidase, and ER stress pathways [9 ^,^ 31]. ER stress leads to the accumulation of unfolded proteins and triggers the onset of UPS comprising three signaling cascades involving protein kinase RNA‐like ER kinase (PERK) protein, IRE1α protein, and activating transcription factor‐6 (ATF6). The harsh oxidative stress microenvironment in degenerated nucleus pulposus cells may further mediate mitochondrial dysfunction [32], extracellular matrix degradation, and cell apoptosis [33]. HGHF induces oxidative stress [34], eventually leading to oxidative damage to macromolecules in cells, especially mitochondria [35]. HGHF treatment significantly promoted oxidative stress and an increase in inflammation. Matrix metalloproteinases are a family of extracellular proteinases and crucial proinflammatory cytokines that degrade the extracellular matrix and aggravate IVD degeneration [36]. By contrast, GPX4 and HO‐1, two important antioxidant enzymes, reportedly reduce ROS levels and mitigate IVD degeneration [37]. Pd NPs profoundly decreased the level of intracellular ROS, reduced AGEs, MMP9, and MMP13 expression, and upregulated GPX4 and HO‐1 levels in HGHF‐stimulated nucleus pulposus cells. Moreover, the evaluation of mitochondria under conditions involving HGHF showed a distinct disturbance in the structure and function of mitochondria in degenerated nucleus pulposus cells. Therefore, Pd NPs protected the structure and function of mitochondria and ameliorated the harsh oxidative stress microenvironment in HGHF‐induced nucleus pulposus cell degeneration.

The ER stress response occurs when the nascent unfolded polypeptide production exceeds the processing capacity of the ER related to mitochondrial function [38] and is associated with the pathogenesis of many diseases, such as diabetes [39]. AGEs accumulation triggers ER stress by activating three central receptors on the surface of the ER: IRE1α, PERK, and ATF6 [40]. Typically, IRE1α splices the mRNA of the transcription factor X‐box binding protein 1, whereas PERK directly phosphorylated the eukaryotic initiation factor 2 subunit, which selectively translates and activates ATF4. ATF6 cleaves itself and induces ER stress [41]. A previous study proposed a strategy to ameliorate IVDD by targeting ER stress [42], highlighting the role of ER stress in AGEs progression caused by IVDD. Our study found that the protein expression of IRE1α and ATF4 was significantly upregulated in the AGEs‐induced nucleus pulposus cell degeneration model. However, activation of these receptors in degenerated nucleus pulposus cells was suppressed after treatment with Pd NPs, suggesting that Pd NPs mediated ER stress amelioration in AGEs‐stimulated nucleus pulposus cells. Pd NPs also effectively inhibited the activated ER stress response and attenuated the AGEs‐induced degeneration model in rat tails.

This study is associated with certain limitations. For the mechanistic investigations, further research is needed to elucidate the basis of structural interaction between Pd NPs and VCP, as well as to explore the enzyme‐like activity of Pd NPs. Second, in terms of application, future studies may benefit from exploring a targetable intravenous application route for Pd NPs, which could avoid multiple direct injections in IVDs. Additionally, while no nanomaterials containing heavy metals have been approved for clinical trials, Pd NPs have demonstrated applications in the treatment of cancer [43] and ulcerative colitis in some pre‐clinical settings [44], suggesting the potential for clinical translation of Pd NPs.

## 4 Conclusion

Pd NPs have been widely used as efficient catalysts and are involved in various chemical transformations, demonstrating good biocompatibility under in vitro and in vivo conditions. In this study, Pd NPs promoted the ATPase activity of VCP and activated the LC3‐mediated autophagy pathways to degrade AGEs, relieve ER stress, and recover mitochondrial function, thereby counteracting the detrimental oxidative stress microenvironment in the IVDD rat model. Consequently, Pd NPs effectively rescued HGHF and AGEs‐induced IVD degeneration in nucleus pulposus cells and animal models. The Pd NPs reported in this study exhibit biocompatibility and can be obtained via a gentle preparation process, showing great potential for clinical translation. Therefore, this study offers an efficient strategy to mitigate IVDD by targeting AGEs clearance via Pd NPs and provides novel insights for developing therapeutic measures for IVDD, especially those associated with DM.

## 5 Materials and Methods

### 5.1 Intervertebral Disc Specimens

Intervertebral discs (≥5 mm) were surgically removed from 15 patients after informed consent was obtained. These tissues were divided into three groups (*n* = 5): the control group consisted of young patients diagnosed with lumbar disc herniation (LDH) (three males and two females with an average age of 23.00 ± 2.236 years; age range: 19 to 24 years). The older group consisted of patients diagnosed with lumbar spinal stenosis (LSS), lumbar disc herniation (LDH), and degenerative lumbar spondylolisthesis (DLS; three males and two females with an average age of 54.80 ± 12.72 years; age range: 34 to 65 years). The DM group consisted of patients diagnosed with lumbar disc herniation (LDH) and lumbar spinal stenosis (LSS; three males and two females with an average age of 65.00 ± 15.98 years; age range: 38 to 79 years; Table S1). The samples were subjected to histological and immunohistochemical analysis. The degree of IVDD was evaluated using the Pfirrmann grading system.

### 5.2 Preparation of Pd NPs

Deionized (DI) water was used in all experiments. All other reagents and chemicals were used as received without further purification. Modified Pd NPs were prepared as previously described [13]. Polyvinylpyrrolidone (3.0 g; average molecular weight ∼55,000; Sigma‐Aldrich; St. Louis, MO, USA) was dissolved in ethylene glycol (5.0 mL) with magnetic stirring. The mixture was then incubated at 160°C in an oil bath with magnetic stirring for 30 min. Na_2_PdCl_4_ solution in ethylene glycol (20.0 mg mL^−1^, 2.0 mL; Sigma‐Aldrich) was mixed, followed by a reaction for 1 h. The mixture was then immersed in an ice‐water bath. The final products were obtained via centrifugation, followed by repeated washing with acetone and DI water. The Pd NPs were then dispersed in DI water.

The Pd NPs were labeled with fluorescent FITC (Energy Chemical). Briefly, 1.0 mL of Pd NPs (10.0 mg mL^−1^) was activated by *N*‐hydroxysuccinimide/1‐ethyl‐3‐(3‐(dimethylamino)propyl) carbodiimide (100 mM; Energy Chemical) for 1 h. Subsequently, 5 mol of NH_2_‐PEG2000‐COOH (Sigma‐Aldrich) was added and allowed to react for 12 h at room temperature (RT). The product was centrifuged, washed, and dispersed with DI water. Finally, 0.2 mL of FITC (1.0 mg mL^−1^) was dispersed, mixed, and stirred at RT overnight. FITC‐labeled PD NPs were then obtained.

### 5.3 Pd NP Characterization

TEM (JEM‐2100F, Japan) was used to analyze particle size, morphology, and elemental mapping. Dynamic light scattering (Zeta Plus, Brookhaven, NY, USA) was used to analyze hydrodynamic size. A UV–Vis–NIR spectrophotometer (UV‐3600, Shimadzu, Japan) was used to analyze ultraviolet‐visible absorption spectra.

### 5.4 VCP ATPase Assay

The reaction buffer was prepared as previously described [24]. Briefly, 500 ng of recombinant active GST‐VCP (Proteintech, Rosemont, IL, USA; #Ag1002) was incubated with 50 µL of Pd NPs (0, 2.5, 5, 10, and 20 µg mL^−1^) or 10 µM DBeQ, NMS873, or CB‐5083 (TargetMol Chemicals, Boston, MA, USA #177355‐84‐9; #1418013‐75‐8; #542705‐92‐9, respectively) in reaction buffer. ATP (1 mM) was added and processed for 1 h at RT. Then, 100 µL phosphate colorimetric assay kit reagent (Sigma‐Aldrich, #MAK030) was added and processed for 30 min at RT. The amount of released phosphate was calculated at an absorbance of 650 nm.

### 5.5 Nucleus Pulposus Cell Cultures

The nucleus pulposus cell line is immortalized [45]. Dulbecco's modified Eagle's medium with 10% fetal bovine serum and 1% penicillin‐streptomycin (Gibco, Thermo Fisher Scientific, Waltham, MA, USA) was used for cell culturing. Cells were cultured at 37°C with 5% CO_2_.

### 5.6 RFP‐GFP‐LC3B Assay

RFP‐GFP‐LC3B (Component A, Premo Autophagy Tandem Sensor RFP‐GFP‐LC3B Kit, Invitrogen Waltham, MA, USA; #P36239) was added to nucleus pulposus cells for 16 h. Subsequently, Pd NPs and NMS873 were introduced for another 16 h to activate autophagy pathways. Finally, the cells were processed using CLSM with Hoechst stain to nuclei.

### 5.7 Seahorse Assay

The seahorse assay was performed following the manufacturer's instructions (Seahorse Bioscience, North Billerica, MA, USA). Nucleus pulposus cells at a density of 5 × 10^3^ cells well^−1^ were treated with Pd NPs, HGHF reagents (200 mM glucose + 300 mM palmitic acid), and AGEs (200 µg mL^−1^), or both for 24 h. In the OCR assay, cells were stimulated with 1.5 µM oligomycin, 2.5 µM FCCP, rotenone, and 0.5 µM antimycin A. Cells were stimulated with 10 mM glucose, 2 µM oligomycin, and 50 mM 2‐DG in extracellular acidification rate assay. Finally, the Seahorse XF‐96 Flux Analyzer was used to evaluate the results.

### 5.8 Intracellular ROS Assay

Nucleus pulposus cells were stimulated with HGHF, HGHF + Pd NPs, AGEs, and AGEs + Pd NPs and incubated with 2′,7′‐dichlorofluorescein diacetate (DCFH‐DA; 10 µM; 30 min). The ROS level was analyzed as per the manufacturer's protocol (Dojindo Laboratories, Kumamoto, Japan). Here, the intensity was evaluated, and IOD/DAPI staining was performed using a Leica DM4000 B epifluorescence microscope (Leica Microsystems, Wetzlar, Germany) and flow cytometry. The results were analyzed using the Image‐Pro Plus 6.0 software (Media Cybernetics, Rockville, MD, USA).

### 5.9 RNA Sequence Assay

Nucleus pulposus cells were treated with Pd NPs (0 or 10 µg mL^−1^; 24 h), and the RNA was extracted and analyzed using the Huada Gene Technology Co., Ltd. (Wuhan, China) sequence platform. Specific analysis methods included volcano plots, KEGG pathway enrichment analysis, heat map analysis, and gene set enrichment analysis according to transcripts per kilobase million. The mechanism and pathway activation involved in Pd NP administration were analyzed on the Mybgi platform (Huada Gene Technology).

### 5.10 WB Assay

Total proteins were isolated and quantified per the manufacturer's protocol (RIPA lysis buffer supplemented with phosphatase and protease inhibitors; M5293, M7528; Abmole, China). Subsequently, equal amounts of proteins (20–30 µg) were processed for electrophoresis and electroblotted onto 0.22‐µm polyvinylidene fluoride membranes (Merck‐Millipore, Burlington, MA, USA) with 5% BSA‐PBS blockade. Membranes were incubated with primary antibodies. After overnight incubation at 4°C (for at least 16 h), membranes were washed and incubated with secondary antibodies (DyLigh 800 4× PEG conjugate; Cell Signaling Technology, Danvers, MA, USA), and fluorescence was detected using a fluorescence imaging system (LI‐COR Odyssey). The following antibodies were included: HO‐1 (cat. no. 10701‐1‐AP; rabbit monoclonal antibody [mAb]), LC3 (cat. no. #4108; rabbit mAb), Beclin‐1 (cat. no. D40C5; rabbit mAb), VCP (cat. no. 10736‐1‐AP; rabbit mAb), GPX4 (cat. no. ab125066; rabbit mAb), AGEs (cat. no. ab176173; rabbit mAb), GPX1 (cat. no. ab22604; rabbit mAb), superoxide dismutase type 1 (SOD1; cat. no. #37385; rabbit mAb), MMP9 (cat. no. ab76003; rabbit mAb), MMP13 (cat. no. ab286191; rabbit mAb), IRE1α (cat. no. 14c10; rabbit mAb), ATF4 (cat. no. #11815; rabbit mAb), P21 (cat. no. ab109199; rabbit mAb), NRF2 (cat. no. 80593‐1‐RR; rabbit mAb), phospho‐eif2α (cat. no. #3398; rabbit mAb), eif2α (cat. no. #5324; rabbit mAb), phospho‐AKT (cat. no. D9E; rabbit mAb), AKT (cat. no. 11E7; rabbit mAb), phospho‐JNK (cat. no. Thr183/Tyr185, G9; rabbit mAb), JNK (rabbit mAb), phospho‐P38 (cat. no. Thr180/Tyr182, D13.14.4E; rabbit mAb), P38 (cat. no. D13E1; rabbit mAb), phospho‐ERK (cat. No. Thr202/Tyr204, and D3F9; rabbit mAb), ERK (cat. No. 137F5; rabbit mAb), and β‐actin (cat. no. D6A8; rabbit mAb).

### 5.11 OXPHOS Assay

Nucleus pulposus cells were stimulated using HGHF and AGEs with or without Pd NPs. Total proteins were isolated, quantified, and boiled at 60°C for 10 min. Equal amounts of proteins (20‐30 µg) were subjected to the aforementioned WB assay with primary antibodies against OXPHOS (cat. no. ab110413; mouse mAb; Abcam, Cambridge, UK).

### 5.12 TEM Assay

Nucleus pulposus cells were treated with HGHF with or without Pd NPs and observed using TEM. Briefly, 2.5% ice‐cold glutaraldehyde was used overnight for cell dissociation and fixation. Osmium tetroxide was used for post‐fixation, and various alcohol concentrations were used for dehydration. Furthermore, propylene oxide and epoxy resin were used for rinsing and impregnation. Uranyl acetate and lead citrate were used as the negative controls. Finally, images were captured using a Hitachi TEM system (HC/HR select = HC − 1, accelerating voltage = 80,000, emission = 10.2, vacuum = 5.6 × 10^−5^ Pa).

### 5.13 Animals and Surgical Procedures

All animal experiments were approved by the Institutional Animal Care and Ethics Committee of the Ninth People's Hospital, Shanghai Jiao Tong University School of Medicine (Shanghai, China) and performed following the principles and procedures of the National Institutes of Health Guide for the Care and Use of Laboratory Animals and the Guidelines for Animal Treatment of Shanghai Jiao Tong University.

Eight‐week‐old Sprague‐Dawley rats (*n* = 6; male) were housed under suitable pathogen‐free conditions. Rats were anesthetized using pentobarbital sodium (5 mg 100 g^−1^ body weight) for surgery. The tails were sterilized and incised to expose the IVD at the coccyx vertebrae 6–9. The IVD at Co6/7 was included as the control group, and the IVD at Co7/8 was treated with AGEs (2 µL; 200 µg mL^−1^) and PBS (2 µL) in the PBS group. The IVDs at Co8/9 were treated with AGEs (2 µL; 200 µg mL^−1^) and Pd NPs (2 µL; 10 µg mL^−1^) in the Pd NPs group. Subsequently, the incision was sutured. After 4 weeks, all rats were euthanized to obtain their tails.

Twelve‐week‐old db/db mice (global knockout of *Lepr* to induce DM in mice, *n* = 3) and paired wt/wt mice (*n* = 3) were purchased from the Gempharmatech Co., Ltd, Jiangsu, China. All mice were euthanized with cervical dislocation, and the spine was harvested.

### 5.14 Biocompatibility Assay

Eight‐week‐old Sprague‐Dawley rats (*n* = 6; male) were housed under suitable pathogen‐free conditions. Rats were anesthetized for surgery using pentobarbital sodium (5 mg 100 g^−1^ body weight). The tails were sterilized and incised to expose the IVD at the coccyx vertebrae 6–9. In the PBS group (*n* = 3), the IVDs Co6/7, Co7/8, and Co8/9 were treated with AGEs (2 µL; 200 µg mL^−1^) and PBS (2 µL). In the Pd NPs group (*n* = 3), these IVDs were treated with AGEs (2 µL; 200 µg mL^−1^) and Pd NPs (2 µL; 10 µg mL^−1^). Subsequently, the incision was sutured.

After 4 weeks, all rats were euthanized. For biochemical analysis, the levels of ALB, TBil, ALT, AST, GLU, γ‐GT, BUN, and Scr were measured in rat serum using the Pointcare V3 automatic biochemical analyzer (Tianjin MNCHIP Technologies Ltd., Tianjin, China) with commercial diagnostic kits. The major organs, including the heart, liver, spleen, lungs, and kidneys, were harvested from the euthanized rats for further histology assay to evaluate the toxicity of Pd NPs.

### 5.15 Histology Assay

IVDs were processed using paraffin and sectioned (thickness: 8 µm) per the manufacturer's protocol. The sections were stained using SO/FG and H&E staining kit (Servicebio, Wuhan, China), and the histological score was calculated as previously described [46].

### 5.16 Immunofluorescence Assay

For IVDs, prepared paraffin sections were incubated in antigen retrieval buffer (Roche Diagnostics) at 37°C for 30 min. After cooling to RT, the sections were washed with PBS, Then, sections were added with an auto‐fluorescence quencher for 5 min and blocked for 30 min with a 0.3% BSA/PBS buffer at RT. Sections were then incubated with primary antibodies at 4°C overnight at 1:100 dilution, including anti‐AGEs (cat. no. ab23722; rabbit mAb; Abcam), anti‐LC3 (cat. no. #4108; rabbit mAb; CST). The next day, sections were washed and then incubated with Alexa Fluor 488 and 555 Conjugate secondary antibody (anti‐rabbit, anti‐mouse, 1:500; Cell Signaling Technology) for 50 min at room temperature in the dark, washed with PBS, and incubated with DAPI solution (Sigma‐Aldrich, St Louis, MO, USA) for 10 min in the dark. Finally, sections were subjected to final washes, air‐drying, and then processed with anti‐fluorescence quenching tablets. Fluorescence images were captured using a Leica DM4000 B epifluorescence microscope (Leica Microsystems), and IOD/DAPI measurements were carried out using Image‐Pro Plus 6.0 software (Media Cybernetics, Inc.).

For the Mito‐tracker, ER tracker, and Golgi tracker assessment of NP cells, cells were seeded onto a confocal dish. At 10% confluence, the cells were stained with live cell stain Hoechst 333 and Mito‐tracker, or ER tracker, or Golgi tracker respectively (cat. no. C1035; cat. no. C1041; cat. no. C1043; Beyotime Technology) at RT for 15 min and then captured using a Leica DM4000 B epifluorescence microscope (*n* = 3 for each group).

For the co‐localization of AGEs and Pd NPs in live NP cells, cells were seeded onto a confocal dish and treated with CY3‐labeled AGEs and FITC‐labeled Pd NPs for 0, 8, 12, and 24 h. After being stained with Hoechst 333 for 14 min, the images of cells were then captured using a Leica DM4000 B epifluorescence microscope (*n* = 3 for each group).

For endocytosis of the Pd NPs in NP cells, cells were seeded and treated with FITC‐Pd NPs (10 µg mL^−1^; 0, 6, 12, and 24 h). At 10% confluence, the cells were fixed with 4% paraformaldehyde, and permeabilized with 0.1% Triton X‐100/PBS solution, then washed with PBS and blocked with 0.3% BSA/PBS buffer for 1 h. Then, cells were processed as sections above for antibodies incubation, of which the primary antibodies were anti‐Rab5 (cat. no. ab218624; rabbit mAb; Abcam) (*n* = 3 for each group).

For AGEs, RAGE, and LC3 assessment of NP cells, cells were seeded and treated respectively. At 10% confluence, the cells were processed as aforementioned, of which the primary antibodies were anti‐AGEs (cat. no. ab23722; rabbit mAb; Abcam), anti‐RAGE (cat. no. ab 228861; rabbit mAb; Abcam), and anti‐LC3 (cat. no. #4108; rabbit mAb) (*n* = 3 for each group).

For Cells, fluorescence images were captured using a Leica DM4000 B epifluorescence microscope (Leica Microsystems), and IOD/DAPI measurements were carried out using Image‐Pro Plus 6.0 software (Media Cybernetics, Inc.).

### 5.17 Immunohistochemistry Assay

The paraffin sections were processed using an immunohistochemistry kit (cat. no. G1215‐200T; Servicebio Technology) according to the manufacturer's instructions. The following primary antibodies were used: anti‐AGEs (cat. no. ab23722; rabbit mAb; Abcam), anti‐RAGE (cat. no. ab 228861; rabbit mAb; Abcam), anti‐LC3 (cat. no. #4108; rabbit mAb), anti‐ATF4 (cat. no. #11815; rabbit mAb), and anti‐VCP (cat. no. 10736‐1‐AP; rabbit mAb). Images were captured using a Leica DM4000 B microscope, and the ratio of the positively‐stained cell of AGEs and RAGE, and the relative IOD of ATF4, LC3, and VCP was calculated using Image Pro Plus 6.0 software.

### 5.18 Radiographic Assay

Digital radiography of the IVDs treated with AGEs with or without Pd NPs was conducted in the anteroposterior axis with a 21 LP mm^−1^ detector and up to 5× geometric magnification (Faxitron VersaVision; Faxitron Bioptics LLC, Tucson, AZ, USA).

### 5.19 MRI Analysis

MRI of discs was captured using the Siemens Magnetom Prisma E11 (Siemens Healthineers, Erlangen, Germany) platform with the following parameters: TR 3000 ms, TE 80 ms, thickness of 1.1 mm, interval of 0.22 mm, FOV of 160 × 65 mm, and voxel size of 0.25 × 0.25 × 1.1 mm.

### 5.20 Statistical Analyses

Data (presented as the mean ± SD) were obtained from three to six independent experiments or repeated measurements. Significant differences among groups were analyzed using one‐way analysis of variance (ANOVA) with Tukey's post‐hoc test or the Kruskal–Wallis test with Dunn's post‐hoc test. All analyses were performed with GraphPad software (version 9.3; NY, USA). Differences were defined as significant with a *p‐*value of < 0.05 (*p < 0.05, **p < 0.01, ***p < 0.001, and ****p < 0.0001).

## Ethics Statement

The human tissues used in the study were extracted as waste material during surgeries performed at the Shanghai Ninth People's Hospital, and written informed consent for the use of the tissues was obtained from the patients (Table S1). Ethics approval for conducting studies on humans was received from the Institutional Human Ethics Review Board of Shanghai Ninth People's Hospital, Shanghai Jiao Tong University School of Medicine (approval no. SH9H‐2021‐T94‐2). Ethics approval for the animal studies was obtained from the Institutional Animal Ethics Review Board of the Shanghai Ninth People's Hospital, Shanghai Jiao Tong University School of Medicine (approval no. SH9H‐2021‐A607‐SB).

## Conflict of Interest Statement

The authors declared no conflicts of interest.

## Supporting information



Supporting information

## Data Availability

The datasets used and/or analyzed during the current study are available from the corresponding author upon reasonable request.

## References

[1] L. Frapin , J. Clouet , V. Delplace , M. Fusellier , J. Guicheux , and C. L.e Visage , “Lessons Learned From Intervertebral Disc Pathophysiology to Guide Rational Design of Sequential Delivery Systems for Therapeutic Biological Factors,” Advanced Drug Delivery Reviews 149–150 (2019): 49. 10.1016/j.addr.2019.08.007 31445063

[2] P. Sampara , R. R. Banala , S. K. Vemuri , A. V. G. Reddy , and G. P. V. Subbaiah , “Understanding the Molecular Biology of Intervertebral Disc Degeneration and Potential Gene Therapy Strategies for Regeneration: A Review,” Gene Therapy 25 (2018): 67. 29567950 10.1038/s41434-018-0004-0

[3] R.‐Z. Yang , W.‐N. Xu , H.‐L. Zheng , et al., “Involvement of Oxidative Stress‐Induced Annulus Fibrosus Cell and Nucleus Pulposus Cell Ferroptosis in Intervertebral Disc Degeneration Pathogenesis,” Journal of Cellular Physiology 236 (2021): 2725. 32892384 10.1002/jcp.30039 PMC7891651

[4] a) L. M. Jaworski , K. L. Kleinhans , and A. R. Jackson , “Effects of Oxygen Concentration and Culture Time on Porcine Nucleus Pulposus Cell Metabolism: An In Vitro Study,” Frontiers in Bioengineering and Biotechnology 7 (2019): 64. 31001527 10.3389/fbioe.2019.00064 PMC6454860

[5] a) P. Zhang , T. Li , X. Wu , E. C. Nice , C. Huang , and Y. Zhang , “Oxidative Stress and Diabetes: Antioxidative Strategies,” Frontiers of Medicine 14 (2020): 583. 32248333 10.1007/s11684-019-0729-1

[6] M. V. Risbud and I. M. Shapiro , “Role of Cytokines in Intervertebral Disc Degeneration: Pain and Disc Content,” Nature Reviews Rheumatology 10 (2014): 44. 24166242 10.1038/nrrheum.2013.160 PMC4151534

[7] Y. Zhao , Q. Xiang , J. Lin , S. Jiang , and W. Li , “Oxidative Stress in Intervertebral Disc Degeneration: New Insights from Bioinformatic Strategies,” Oxidative Medicine and Cellular Longevity 2022 (2022): 2239770. 35401932 10.1155/2022/2239770 PMC8991415

[8] F. Cannata , G. Vadala , L. Ambrosio , et al., “Intervertebral Disc Degeneration: A Focus on Obesity and Type 2 Diabetes,” Diabetes Metabolism Research and Reviews 36 (2020): e3224. 31646738 10.1002/dmrr.3224

[9] C. Zeng , Y. Li , J. Ma , L. Niu , and F. R. Tay , “Clinical/Translational Aspects of Advanced Glycation End‐Products,” Trends in Endocrinology and Metabolism 30 (2019): 959. 31597608 10.1016/j.tem.2019.08.005

[10] R. Luo , S. Li , G. Li , et al., “FAM134B‐Mediated ER‐Phagy Upregulation Attenuates AGEs‐Induced Apoptosis and Senescence in Human Nucleus Pulposus Cells,” Oxidative Medicine and Cellular Longevity 2021 (2021): 3843145. 34394825 10.1155/2021/3843145 PMC8363461

[11] J. Wang , B. Cheng , J. Li , et al., “Chemical Remodeling of Cell‐Surface Sialic Acids Through a Palladium‐Triggered Bioorthogonal Elimination Reaction,” Angewandte Chemie International Edition 54 (2015): 5364. 25765364 10.1002/anie.201409145

[12] E. Indrigo , J. Clavadetscher , S. V. Chankeshwara , A. Lilienkampf , and M. Bradley , “Palladium‐Mediated In Situ Synthesis of an Anticancer Agent,” Chemical Communications 52 (2016): 14212. 27886301 10.1039/c6cc08666g

[13] Y. Wang , A. Biby , Z. Xi , B. Liu , Q. C. Rao , and X. H. Xia , “One‐Pot Synthesis of Single‐Crystal Palladium Nanoparticles With Controllable Sizes for Applications in Catalysis and Biomedicine,” ACS Applied Nano Materials 2 (2019): 4605.

[14] H. J. Lee , Y. H. Jung , G. E. Choi , et al., “Urolithin a Suppresses High Glucose‐Induced Neuronal Amyloidogenesis by Modulating TGM2‐Dependent ER‐Mitochondria Contacts and Calcium Homeostasis,” Cell Death and Differentiation 28 (2021): 184. 32704090 10.1038/s41418-020-0593-1 PMC7852667

[15] T. Grunhagen , G. Wilde , D. M. Soukane , S. A. Shirazi‐Adl , and J. P. G. Urban , “Nutrient Supply and Intervertebral Disc Metabolism,” Journal of Bone and Joint Surgery. American Volume 88 (2006): 30. 16595440 10.2106/JBJS.E.01290

[16] K. Alpantaki , A. Kampouroglou , C. Koutserimpas , G. Effraimidis , and A. Hadjipavlou , “Diabetes Mellitus as a Risk Factor for Intervertebral Disc Degeneration: A Critical Review,” European Spine Journal 28 (2019): 2129. 31201565 10.1007/s00586-019-06029-7

[17] Z. Zhang , J. Lin , M. Nisar , et al., “The sirt1/P53 axis in diabetic intervertebral disc degeneration pathogenesis and therapeutics,” Oxidative Medicine and Cellular Longevity 2019 (2019): 7959573. 31583043 10.1155/2019/7959573 PMC6754956

[18] C. Cunha , A. J. Silva , P. Pereira , R. Vaz , R. M. Gonçalves , and M. A. Barbosa , “The Inflammatory Response in the Regression of Lumbar Disc Herniation,” Arthritis Research & Therapy 20 (2018): 251. 30400975 10.1186/s13075-018-1743-4 PMC6235196

[19] F. Barutta , S. Bellini , S. Kimura , et al., “Protective Effect of the Tunneling Nanotube‐TNFAIP2/M‐Sec System on Podocyte Autophagy in Diabetic Nephropathy,” Autophagy 19 (2023): 505. 35659195 10.1080/15548627.2022.2080382 PMC9851239

[20] S. Ashe , D. Nayak , M. Kumari , and B. Nayak , “Ameliorating Effects of Green Synthesized Silver Nanoparticles on Glycated End Product Induced Reactive Oxygen Species Production and Cellular Toxicity in Osteogenic Saos‐2 Cells,” ACS Applied Materials & Interfaces 8 (2016): 30005. 27749032 10.1021/acsami.6b10639

[21] a) J. Xu , L. You , and Z. Zhao , “Synthesize of the Chitosan‐Tpp Coated Betanin‐Quaternary Ammonium‐Functionalized Mesoporous Silica Nanoparticles and Mechanism for Inhibition of Advanced Glycation End Products Formation,” Food Chemistry 407 (2023): 135110. 36495745 10.1016/j.foodchem.2022.135110

[22] a) Z. Cai , Y. Li , L. Bai , et al., “Tetrahedral Framework Nucleic Acids Based Small Interfering RNA Targeting Receptor for Advanced Glycation End Products for Diabetic Complications Treatment,” ACS Nano 17 (2023): 22668. 37751401 10.1021/acsnano.3c06999

[23] Z. Ye , C. Hu , J. Wang , et al., “Burst of hopping Trafficking Correlated Reversible Dynamic Interactions Between Lipid Droplets and Mitochondria Under Starvation,” Exploration 3 (2023): 20230002. 37933279 10.1002/EXP.20230002 PMC10582609

[24] L. Wrobel , S. M. Hill , A. Djajadikerta , et al., “Compounds Activating VCP D1 ATPase Enhance Both Autophagic and Proteasomal Neurotoxic Protein Clearance,” Nature Communications 13 (2022): 4146. 10.1038/s41467-022-31905-0 PMC9288506 35842429

[25] a) D. Ritz , M. Vuk , P. Kirchner , et al., “Endolysosomal Sorting Oof Ubiquitylated Caveolin‐1 is Regulated by VCP and UBXD1 and Impaired by VCP Disease Mutations,” Nature Cell Biology 13 (2011): 1116. 21822278 10.1038/ncb2301 PMC3246400

[26] J. Fielden , M. Popović , and K. Ramadan , “TEX264 at the Intersection of Autophagy and DNA Repair,” Autophagy 18 (2022): 40. 33726628 10.1080/15548627.2021.1894059 PMC8865260

[27] R.‐Q. Yao , C. Ren , Z.‐F. Xia , and Y.‐M. Yao , “Organelle‐Specific Autophagy in Inflammatory Diseases: A Potential Therapeutic Target Underlying the Quality Control Of Multiple Organelles,” Autophagy 17 (2021): 385. 32048886 10.1080/15548627.2020.1725377 PMC8007140

[28] K. Nowotny , D. Schröter , M. Schreiner , and T. Grune , “Dietary Advanced Glycation End Products and Their Relevance for Human Health,” Ageing Research Reviews 47 (2018): 55. 29969676 10.1016/j.arr.2018.06.005

[29] M. B. Manigrasso , P. Rabbani , L. Egaña‐Gorroño , et al., “Small‐Molecule Antagonism of the Interaction of the Rage Cytoplasmic Domain with Diaph1 Reduces Diabetic Complications in Mice,” Science Translational Medicine 13 (2021): eabf7084. 34818060 10.1126/scitranslmed.abf7084 PMC8669775

[30] a) C. Nie , Y. Li , H. Qian , H. Ying , and L. Wang , “Advanced Glycation End Products in Food and Their Effects on Intestinal Tract,” Critical Reviews in Food Science and Nutrition 62 (2022): 3103. 33356474 10.1080/10408398.2020.1863904

[31] B. I. Hudson and M. E. Lippman , “Targeting RAGE Signaling in Inflammatory Disease,” Annual Review of Medicine 69 (2018): 349. 10.1146/annurev-med-041316-085215 29106804

[32] Z. Tang , B. Hu , F. Zang , J. Wang , X. Zhang , and H. Chen , “Nrf2 Drives Oxidative Stress‐Induced Autophagy in Nucleus Pulposus Cells via a Keap1/Nrf2/p62 Feedback Loop to Protect Intervertebral Disc From degeneration,” Cell Death & Disease 10 (2019): 510. 31263165 10.1038/s41419-019-1701-3 PMC6602960

[33] K. Sun , X. Jing , J. Guo , X. Yao , and F. Guo , “Mitophagy in Degenerative Joint Diseases,” Autophagy 17 (2021): 2082. 32967533 10.1080/15548627.2020.1822097 PMC8496714

[34] b) A. Singh , R. Kukreti , L. Saso , and S. Kukreti , “Mechanistic Insight into Oxidative Stress‐Triggered Signaling Pathways and Type 2 Diabetes,” Molecules 27 (2022): 950. 35164215 10.3390/molecules27030950 PMC8840622

[35] C. Carvalho and S. Cardoso , “Diabetes–Alzheimer's Disease Link: Targeting Mitochondrial Dysfunction and Redox Imbalance,” Antioxidants & Redox Signaling 34 (2021): 631. 32098477 10.1089/ars.2020.8056

[36] T. Zhou , X. Yang , Z. Chen , et al., “Prussian Blue Nanoparticles Stabilize SOD1 From Ubiquitination‐Proteasome Degradation to Rescue Intervertebral Disc Degeneration,” Advanced Science 9 (2022): e2105466. 35128840 10.1002/advs.202105466 PMC8981911

[37] X. Yang , Y. Chen , J. Guo , et al., “Polydopamine Nanoparticles Targeting Ferroptosis Mitigate Intervertebral Disc Degeneration via Reactive Oxygen Species Depletion, Iron Ions Chelation, and GPX4 Ubiquitination Suppression,” Advanced Science 10 (2023): e2207216. 36951540 10.1002/advs.202207216 PMC10161035

[38] Y. Li , D. Huang , and L. Jia , et al., “LonP1 Links Mitochondria‐ER Interaction to Regulate Heart Function,” Research 6 (2023): 0175. 37333972 10.34133/research.0175 PMC10275618

[39] B. Basha , S. M. Samuel , C. R. Triggle , and H. Ding , “Endothelial Dysfunction in Diabetes Mellitus: Possible Involvement of Endoplasmic Reticulum Stress?,” Experimental Diabetes Research 2012 (2012): 481840. 22474423 10.1155/2012/481840 PMC3299342

[40] a) D. T. Rutkowski and R. J. Kaufman , “A Trip to the ER: Coping With stress,” Trends in Cell Biology 14 (2004): 20. 14729177 10.1016/j.tcb.2003.11.001

[41] a) J. H. Lin , H. Li , D. Yasumura , et al., “IRE1 Signaling Affects Cell Fate During the Unfolded Protein Response,” Science 318 (2007): 944. 17991856 10.1126/science.1146361 PMC3670588

[42] Z. Liao , R. Luo , G. Li , et al., “Exosomes From Mesenchymal Stem Cells Modulate Endoplasmic Reticulum Stress to Protect Against Nucleus Pulposus Cell Death and Ameliorate Intervertebral Disc Degeneration In Vivo,” Theranostics 9 (2019): 4084. 31281533 10.7150/thno.33638 PMC6592170

[43] M. A. Miller , B. Askevold , H. Mikula , R. H. Kohler , D. Pirovich , and R. Weissleder , “Nano‐palladium is a Cellular Catalyst for In Vivo Chemistry,” Nature Communications 8 (2017): 15906. 10.1038/ncomms15906 PMC5510178 28699627

[44] J. Cheng , Y. Zhang , L. Ma , et al., “Macrophage‐Derived Extracellular Vesicles‐Coated Palladium Nanoformulations Modulate Inflammatory and Immune Homeostasis for Targeting Therapy of Ulcerative Colitis,” Advanced Science 10 (2023): e2304002. 37807805 10.1002/advs.202304002 PMC10667822

[45] C.‐d. Oh , H.‐J. Im , J. Suh , A. Chee , H. An , and D. Chen , “Rho‐Associated Kinase Inhibitor Immortalizes Rat Nucleus Pulposus and Annulus Fibrosus Cells,” Spine 41 (2016): E255. 26693672 10.1097/BRS.0000000000001235 PMC4769661

[46] M.‐L. Ji , H. Jiang , X.‐J. Zhang , et al., “Preclinical Development of a MicroRNA‐Based Therapy for Intervertebral Disc Degeneration,” Nature Communications 9 (2018): 5051. 10.1038/s41467-018-07360-1 PMC6262020 30487517

