# Supplementary material for: Palladium Nanoparticles Degrade Advanced Glycation End Products via Valosin‐Containing Protein Mediated Autophagy to Attenuate High‐Glucose/High‐Fat‐Induced Intervertebral Disc Degeneration
Source: Exploration (Beijing). 2025 Jan 17;5(2):20230174. doi: 10.1002/EXP.20230174 (PMC12087406; doi:10.1002/EXP.20230174)
Supplement: Supplementary file 1 — Supporting information [file EXP2-5-20230174-s001.docx]

**Supplementary Information**

**Palladium Nanoparticles Degrade Advanced Glycation End Products via Valosin-Containing Protein Mediated Autophagy to Attenuate High-glucose/High-fat Induced Intervertebral Disc Degeneration**

- **Short title: Pd NPs mitigate IVDD via autophagy towards AGEs**

Xiao Yang^1^†, Xiankun Cao^1^†, Xin Wang^1^†, Jiadong Guo^1^, Yangzi Yang^2^, Liqiang Lu^3^, Pu Zhang^1^, Huan Yang^1, 4^, Kewei Rong^1^, Tangjun Zhou^1^, Yongqiang Hao^1^, Jie Zhao^1^*, Jingke Fu^1^*, Kai Zhang^1^*

^1^Shanghai Key Laboratory of Orthopedic Implants, Department of Orthopedics, Ninth People’s Hospital, Shanghai Jiao Tong University School of Medicine, 639 Zhizaoju Road, Shanghai, 200011, China

^2^Department of Orthopedic Surgery, Spine Center, Changzheng Hospital, Navy Medical University, No. 415 Fengyang Road, Shanghai 200003, China

^3^Institute of Electrochemical Energy Storage, Helmholtz-Zentrum Berlin für Materialien und Energie, Hahn-Meitner-Platz 1, 14109 Berlin, Germany

^4^The Second Clinical Medical College of Yunnan University of Traditional Chinese Medicine, 1076 Yuhua Road, Kunming, 650500, China

***Correspondence:**

Kai Zhang: orth_kai@163.com

Jingke Fu: fujingke@sjtu.edu.cn

Jie Zhao: profzhaojie@126.com

†These authors contributed equally to this work





**Figure. S1.** (A) Quantifying the Pfirrmann grade in patients shown in Table 1. All data are presented as mean ± standard deviation (SD) from five replicates. ^*^*p*<0.05, ^**^*p*<0.01, ^***^*p*<0.001, and ^****^*p*<0.0001.


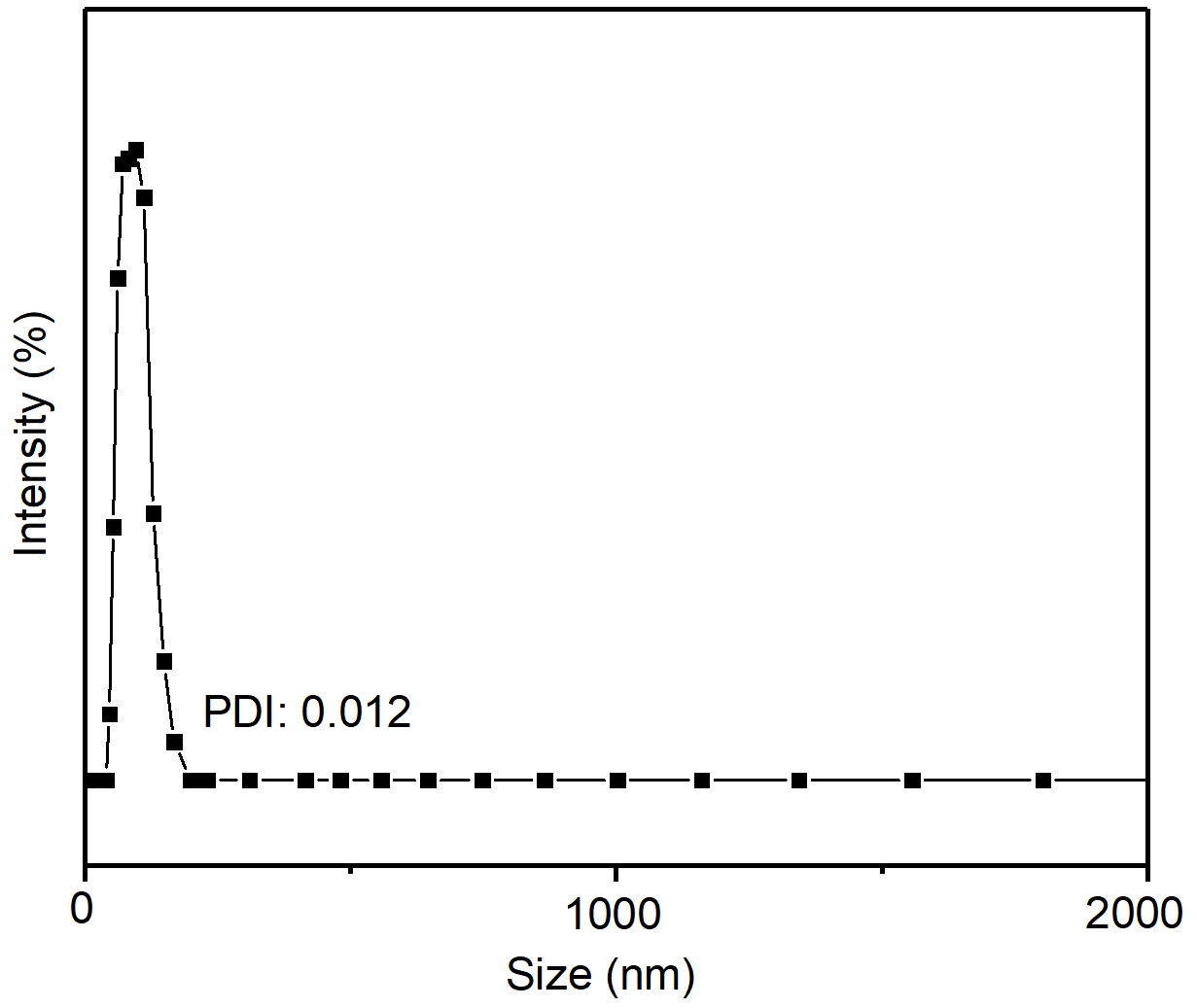


**Figure. S2.** The hydrodynamic particle size distribution of Pd NPs after incubating in PBS medium for 14 days.



**Figure. S3.** (A) Immunofluorescence analysis of Cy3-AGEs and FITC-Pd NPs in NP cells stimulated with Cy3 labeled advanced glycation end products (AGEs) (200 μg mL^-1^) for 0, 8, 12, and 24 h with or without FITC-Pd NPs for 24 h. (B) Integrated optical density (IOD)/DAPI quantification of Cy3-AGEs as shown in A. All data are presented as mean ± standard deviation (SD) from three replicates. ^*^*p*<0.05, ^**^*p*<0.01, ^***^*p*<0.001, and ^****^*p*<0.0001.



**Figure. S4.** (A) Immunofluorescence analysis of receptor for advanced glycation endproduct (RAGE) in nucleus pulposus (NP) cells stimulated with FITC-Pd NPs (10 μg mL^-1^), high-glucose/high-fat (HGHF), and HGHF/FITC-Pd NPs for 24 h. (B) Integrated optical density (IOD)/DAPI quantification of RAGE shown in A. All data are presented as mean ± standard deviation (SD) from three replicates. ^*^*p*<0.05, ^**^*p*<0.01, ^***^*p*<0.001, and ^****^*p*<0.0001.





**Figure. S5.** (A) Integrated optical density (IOD)/DAPI quantification of LC3 as shown in Figure 3F. (B) Integrated optical density (IOD)/DAPI quantification of LC3 as shown in Figure 3M. All data are presented as mean ± standard deviation (SD) from five replicates. ^*^*p*<0.05, ^**^*p*<0.01, ^***^*p*<0.001, and ^****^*p*<0.0001.



**Figure. S6.** (A) Immunofluorescence analysis of 2′,7′-dichlorofluorescein diacetate (DCFH-DA) in nucleus pulposus (NP) cells stimulated with Pd NPs, advanced glycation end products (AGEs) (200 μg mL^-1^), and AGEs/Pd NPs for 24 h. (B) Quantifying relative fluorescence intensity of DCFH-DA shown in A. (C) Flow cytometry analysis of NP cells stained with DCFH-DA and stimulated with Pd NPs, AGEs, and AGEs/Pd NPs for 24 h. All data are presented as mean ± standard deviation (SD) from three replicates. ^*^*p*<0.05, ^**^*p*<0.01, ^***^*p*<0.001, and ^****^*p*<0.0001.



**Figure. S7.** (A) Oxygen consumption rate (OCR) assay of nucleus pulposus (NP) cells stimulated with Pd NPs, advanced glycation end products (AGEs), and AGEs/Pd NPs for 24 h. (B) Quantifying basal respiration, maximal respiration, and spare respiratory capacity shown in A. (C) Extracellular acidification rate (ECAR) assay of NP cells stimulated with Pd NPs, AGEs, and AGEs /Pd NPs for 24 h. (D) Quantifying glycolysis, glycolytic capacity, and glycolytic reserve shown in C. All data are presented as mean ± standard deviation (SD) from three replicates. ^*^*p*<0.05, ^**^*p*<0.01, ^***^*p*<0.001, and ^****^*p*<0.0001.


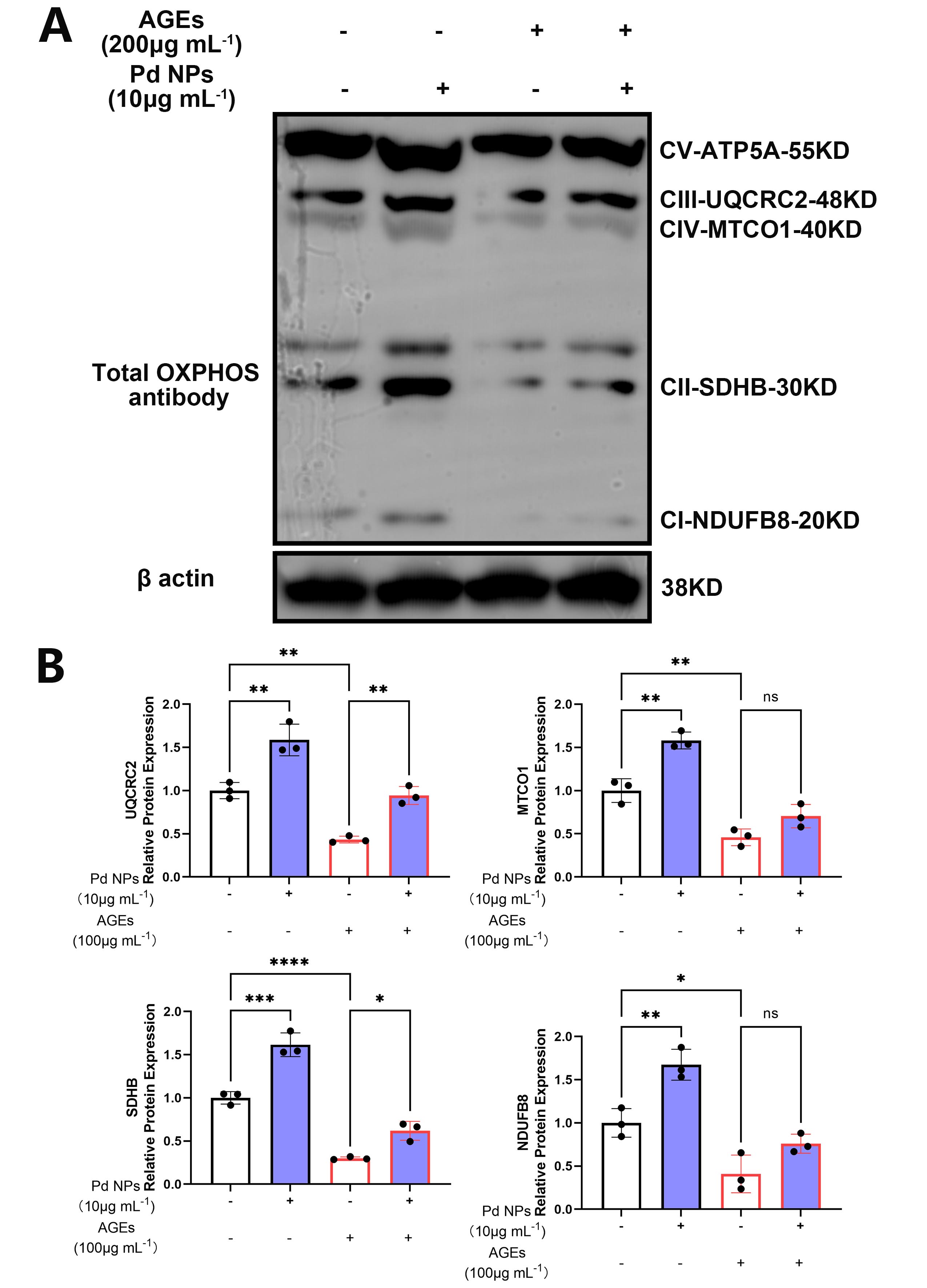
**Figure. S8.** (A) Western blot analysis of total oxidative phosphorylation (OXPHOS) proteins in nucleus pulposus (NP) cells stimulated with Pd NPs, advanced glycation end products (AGEs), and AGEs/Pd NPs for 24 h. (B) Semi-quantifying grayscale value in UQCRC2, MTCO1, SDHB, and NDUFB8 in A. All data are presented as mean ± standard deviation (SD) from three replicates. ^*^*p*<0.05, ^**^*p*<0.01, ^***^*p*<0.001, and ^****^*p*<0.0001.





**Figure. S9.** (A) Biochemical analyses of the levels of albumin (ALB), total bilirubin (TBil), alanine aminotransferase (ALT), aspartate aminotransferase (AST), glucose (GLU), gamma-glutamyltransferase (γ-GT), urea nitrogen (BUN), and creatinine (Scr) in rat serum. (B) H&E staining of the major organs including heart, liver, spleen, lung, and kidney. All data are presented as mean ± SD from three replicates. *p<0.05, **p<0.01, ***p<0.001, and ****p<0.0001.

**Table S1.** **Patient Information**

| Name | Pfirrmann Grade | Gender | Age | Diagnosis | Dibetes Mellitus |
| --- | --- | --- | --- | --- | --- |
| Liu | 5 | female | 38 | LDH | DM |
| Shen | 4 | male | 74 | LSS | DM |
| Liu | 5 | male | 65 | LDH | DM |
| Jiang | 4 | female | 79 | LSS | DM |
| Chen | 4 | male | 69 | LDH | DM |
|  |  |  |  |  |  |
| Guo | 4 | Female | 59 | LSS | Non-DM |
| Zhou | 3 | male | 34 | LDH | Non-DM |
| Wu | 5 | male | 52 | LSS | Non-DM |
| Wu | 4 | female | 64 | DLS | Non-DM |
| Weng | 5 | male | 65 | LSS | Non-DM |
|  |  |  |  |  |  |
| Fang | 2 | male | 19 | LDH | Non-DM |
| Li | 3 | male | 24 | LDH | Non-DM |
| Lin | 2 | female | 24 | LDH | Non-DM |
| Huang | 3 | male | 24 | LDH | Non-DM |
| Yang | 2 | female | 24 | LDH | Non-DM |
